# Early Detection of Red Palm Weevil Infestation: A Systematic Review of Sensing Technologies, Artificial Intelligence, and Field Readiness

**DOI:** 10.3390/insects17101081

**Published:** 2026-10-19

**Authors:** Lubna A. Gabralla, Amnah Alshahrani

**Affiliations:** 1 Department of Computer Science, Applied College, Princess Nourah bint Abdulrahman University, Riyadh 11671, Saudi Arabia; 2 Department of Information Systems, College of Computer and Information Sciences, Princess Nourah bint Abdulrahman University, Riyadh 11671, Saudi Arabia; aaalshahrani@pnu.edu.sa

**Keywords:** early detection, red palm weevil, artificial intelligence, acoustic sensing, thermal imaging, Internet of Things, multimodal fusion, explainable artificial intelligence, plant protection

## Abstract

The red palm weevil (*Rhynchophorus ferrugineus* Olivier; RPW) is an economically important invasive pest of palms, and early detection remains challenging because larval infestation develops internally before visible symptoms appear. This systematic review evaluates current RPW detection technologies, their operational limitations, and priorities for future development. Following PRISMA 2020, 56 primary studies were included, principally covering the period from 2015 to July 2026, together with selected seminal earlier studies. The evidence base was established through a systematic search across five bibliographic and scholarly sources, complemented by a structured supplementary update and source-verification procedure covering publications through 25 July 2026. Acoustic and vibroacoustic sensing provided the most direct evidence of active internal larval activity, whereas thermal, spectral, RGB, and UAV-based approaches primarily detected host physiological stress or externally observable damage. Although several studies reported high classification performance, most evidence was derived from private, single-site, proxy, or controlled datasets, with limited external field validation and inconsistent evaluation protocols. Multimodal and IoT-enabled systems showed potential for continuous and scalable monitoring, but consistent superiority over matched unimodal baselines was not established. No included study explicitly implemented and quantitatively evaluated a recognised explainable artificial intelligence method. The review also identifies major benchmarking and reproducibility limitations and proposes a conceptual six-layer weather-aware multimodal AI framework to guide the development of environmentally robust and field-deployable RPW monitoring systems.

## Simple Summary

The red palm weevil damages palm trees from the inside, so infestations may remain hidden until serious damage has occurred. We reviewed 56 research studies to assess how sensors and computer programmes can help detect and monitor this pest. Sound and vibration sensors provided the most direct evidence of young insects feeding inside palms. Heat-sensitive cameras and other imaging methods mainly revealed changes in tree condition or visible damage, while traps monitored adult insects. Although some methods achieved high performance in individual studies, many were tested using limited data or under controlled conditions, with little testing in other orchards. Combining different sensors may support continuous monitoring, but clear benefits over individual sensors have not yet been established. We identify the need for shared research data and testing across orchards, seasons, and weather conditions. We also outline a proposed system combining sensor readings and weather information, which still requires practical testing. More reliable detection could help growers identify affected trees sooner, protect valuable palm crops, and target treatment more effectively.

## 1. Introduction

### 1.1. Global Importance of Date Palm and the Challenge of Early RPW Detection

The red palm weevil, *Rhynchophorus ferrugineus* Olivier (Coleoptera: Curculionidae), is an invasive pest of great economic importance that threatens palm trees. This pest originated in South and Southeast Asia, and its range expanded as a result of the transfer of infested agricultural materials. Currently, it is found in large areas of Asia, the Middle East, North Africa, Europe, and other regions [1].

The red palm weevil causes major economic and phytosanitary problems in date-producing regions. In Saudi Arabia, annual expenditures on RPW control and treatment programmes are estimated at approximately USD 34.4 million, while the net present value of losses from fallen palms and associated forgone revenues is estimated at approximately USD 401 million annually [2]. In Spain, Italy, and France, the total costs of pest management, elimination, and replacement of affected trees, together with associated losses, were approximately €90 million by 2013 and were projected to reach €200 million by 2023 without effective containment measures [3].

The main difficulty in detecting red palm weevil infestation arises from the concealed development of its larvae within palm tissues. Early-stage infestations may produce no obvious external signs, while visible symptoms generally become more evident as internal damage progresses [1]. Visual inspection uses observable damage symptoms to locate infested palms, whereas pheromone traps monitor adult captures and help prioritise palms for subsequent inspection [1]. Acoustic detection, thermal imaging, and density-based methods, by contrast, are generally applied at the individual-palm level [4,5].

**Figure 1:**
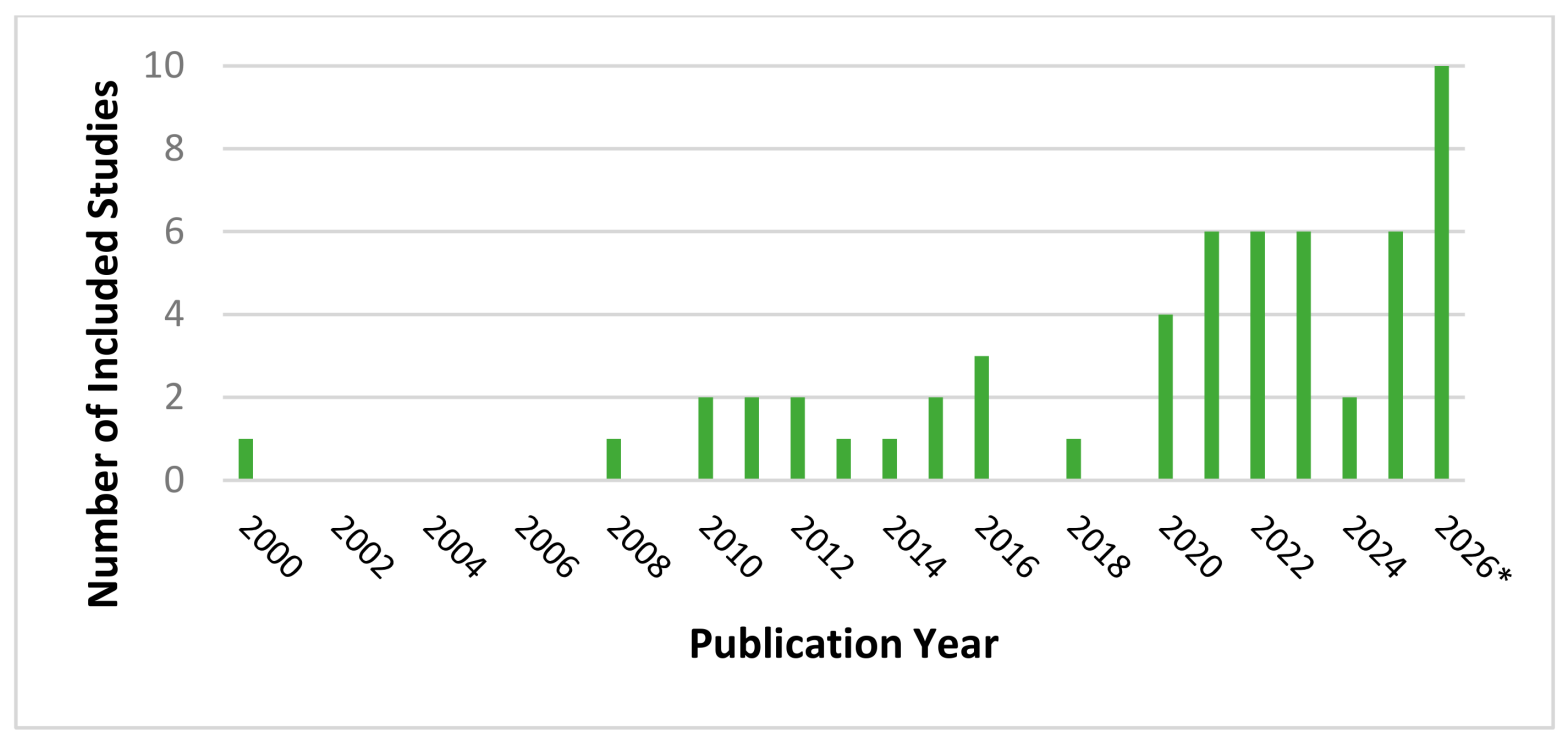
Publication–year distribution of the 56 primary studies included in this review. * The 2026 count includes studies published or available online up to 25 July 2026.

Evidence from the Al-Ahsa Oasis shows that intensified area-wide management combining RPW monitoring, pesticide control, pheromone trapping, and removal of heavily infested palms reduced the infestation rate from 3.7% to 0.36% over three years [6]. Therefore, the primary challenge is not a lack of control methods, but rather detecting infestations before extensive internal damage develops. This need has encouraged growing interest in the development of reliable early-detection technologies. Figure 1 illustrates the clear increase in the number of studies published after 2020, with this growth focusing on artificial intelligence, interconnected sensing technologies, and multimodal monitoring.

### 1.2. Biological Basis of Early RPW Detection

The biological basis of RPW detection is closely linked to its developmental cycle, which comprises egg, multiple larval instars, pupal, and adult stages. Following egg hatch, larvae develop and feed within palm tissues, where substantial internal damage may occur before external symptoms become apparent. The duration and progression of development are influenced by environmental conditions, particularly temperature [7]. Consequently, “early detection” is more appropriately interpreted in relation to biological stage and the signal available for measurement than as a fixed number of days after infestation.

Different developmental stages generate different opportunities for detection. Active larval feeding and movement can generate acoustic and vibroacoustic signals detectable before visible symptoms appear [4], whereas thermal methods depend on infestation-associated physiological responses of the host [5]. Visual inspection generally becomes informative after external symptoms develop, while pheromone trapping targets free-flying adults and therefore reflects population activity rather than concealed infestation within a specific palm [8]. These biological differences provide the basis for the stage-oriented comparison presented later in Section 9.2.

### 1.3. Evolution and Taxonomy of RPW Detection Technologies

Detection methods for the red palm weevil have shifted from traditional approaches heavily reliant on operator expertise to technology-assisted approaches, including combinations of medical, computer, and electronic technologies targeting the larval stage, as well as sensor-based and automated techniques. For example, sensing technologies are classified according to the type of biological or physical signal they measure, such as acoustic methods and internal vibrations resulting from larval feeding and movement [9,10]. RGB systems, multispectral imaging, and drones measure visual or spectral indicators and can be applied at the individual palm or orchard level [11,12], whereas thermal imaging detects temperature changes and physiological functions associated with infestation [5,12]. Template-based image processing has also been used to recognise adult RPW in images. Biohybrid sensing targets chemical compounds associated with the red palm weevil [13]. Microwave and dielectric measurement techniques search for internal structural features or electromagnetic signals associated with infestation [14,15]. Distributed acoustic sensing also enables the monitoring of vibrations across a wide spatial area encompassing multiple palm trees [10,16].

**Figure 2:**
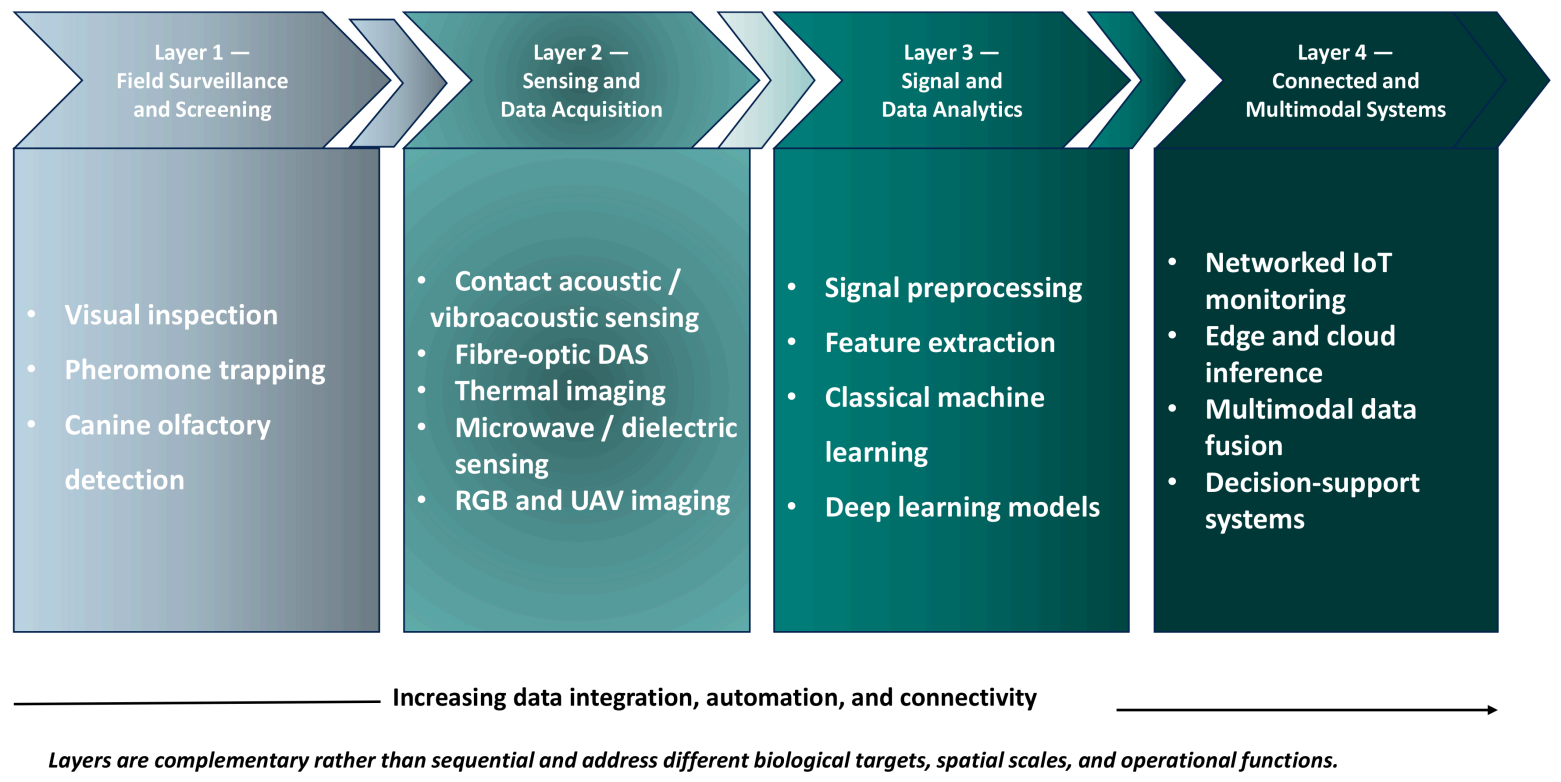
Layered conceptual framework for RPW detection and surveillance. Arrows indicate increasing data integration, automation, and connectivity across complementary layers.

Sensing is currently being integrated with a growing range of analytics and computing technologies. These include rule-based systems, machine learning, deep learning, and wireless connectivity. They also include edge or cloud-based Internet of Things (IoT) platforms. Multimodal systems aim to collect integrated signals within a unified monitoring framework. However, their operational viability depends on several key requirements. These include a reliable biological reference, clear data sources, and validation in outdoor field environments. They must also demonstrate superiority over single-mode baselines evaluated under equivalent conditions. Section 3, Section 4, Section 5, Section 6 and Section 7 examine these technology families, analytical approaches, and integration strategies in greater detail. Figure 2 presents a layered conceptual framework spanning field surveillance and screening, sensing and data acquisition, signal and data analytics, and connected multimodal systems. The layers are complementary rather than sequential and differ in biological target, spatial scale, automation, and degree of system integration.

### 1.4. Objectives and Contributions of This Review

Based on the PRISMA 2020 guidelines [17], this systematic review addresses these challenges through a structured analysis of the evidence. It includes selected studies published before 2015 because they introduced foundational RPW detection techniques or provided quantitative validation of such approaches. The review focuses primarily on peer-reviewed studies published between 2015 and July 2026 and distinguishes itself from previous reviews through five key contributions [1,18,19]:

- Analysis of the impact of environmental factors: The review gathers evidence regarding the impact of environmental conditions on red palm weevil detection performance. These factors include wind speed, ambient temperature, solar load, and humidity. These effects are assessed across acoustic, thermal, visual, and trap-based methods.
- AI and machine learning analysis: The review examines machine learning and deep learning models without limiting itself to a single type of sensing. It includes acoustic, thermal, visual, and multimodal data related to the red palm weevil. This approach allows for the systematic synthesis of evidence across different modes. However, published performance metrics cannot be directly compared due to differences in biological targets, datasets, and validation designs.
- The Explainable AI Gap: This review assesses the limitations of using explainable artificial intelligence (XAI) techniques in red palm weevil detection research. It identifies this limitation as a methodological gap and discusses the potential for integrating model interpretation techniques, such as Grad-CAM and SHAP.
- Structured Research Gaps Framework: This review presents a research gap framework, RG-1 to RG-8. This framework compiles the key scientific, technical, and application challenges identified by the studies reviewed. It also outlines future research priorities needed to advance red palm weevil detection techniques.
- Evaluating Datasets: This review examines public and proprietary datasets used in red palm weevil detection studies across different sensing modes. It also highlights current limitations related to standardising datasets, reproducibility of results, and the development of multimodal models.

The remainder of this paper is organised as follows. Section 2 defines the PRISMA 2020-based systematic review methodology, including the search strategy, study-selection process, and eligibility criteria. Section 3, Section 4, Section 5, Section 6 and Section 7 review conventional detection methods, acoustic and vibroacoustic detection methods, thermal, RGB, UAV and other imaging approaches, artificial intelligence, machine learning and IoT-enabled detection, and multimodal detection and the proposed weather-aware framework, respectively. Section 8 examines datasets, benchmark resources, and evaluation limitations in RPW detection research. Section 9 presents a comparative analysis of the reviewed technologies. Section 10 discusses the research gaps and future research directions. Section 11 concludes the review and presents the recommendations.

## 2. Review Methodology

### 2.1. Review Design, Protocol, and Research Questions

This systematic review was described according to the PRISMA 2020 statement [17]. The protocol was developed before the literature search but was not prospectively registered. The eligibility criteria and planned narrative synthesis approach were specified before screening began. A retrospective, time-stamped protocol record was deposited on the Open Science Framework on 5 August 2026, after study selection and data extraction were complete [20]. The review focused on three key research questions:

1. What techniques and methodologies have been studied for the detection or monitoring of red palm weevil (*Rhynchophorus ferrugineus*) infestations?
2. What performance, validation, and applicability characteristics are reported for traditional, acoustic, and imaging methods, artificial intelligence and machine learning techniques, IoT-enabled systems, and multimodal approaches?
3. What are the remaining scientific, methodological and operational gaps?

The main eligibility period included studies published from 2015 to 25 July 2026. A few studies prior to 2015 were also retained. These were confined to studies that described a basic method for detecting the red palm weevil or that were underpinned by quantitative validation. It was also essential that these studies be relevant to the comparative classification used in the review.

### 2.2. Information Sources and Search Strategy

The initial online search was conducted in May 2026. It included the Web of Science Core Collection, IEEE Xplore, ScienceDirect, Scopus, and Google Scholar. The search strategy was based on three main conceptual groups. The first group addressed the target pest: *Rhynchophorus ferrugineus*, the red palm weevil, abbreviated as RPW. The second group focused on the detection objective, including detection, monitoring, identification, and early warning. The third group addressed the technology modalities, including acoustic and vibrational sensing, machine learning, deep learning, computer vision, thermal and spectral imaging, IoT, sensor fusion, and multimodal monitoring. Search syntax was adapted to each database.

The initial search yielded 129 records. These included 38 from Web of Science, 31 from Scopus, 19 from IEEE Xplore, 14 from ScienceDirect and 27 from Google Scholar. Details of search strings and filters for each source are provided in Supplementary Table S2.

**Figure 3:**
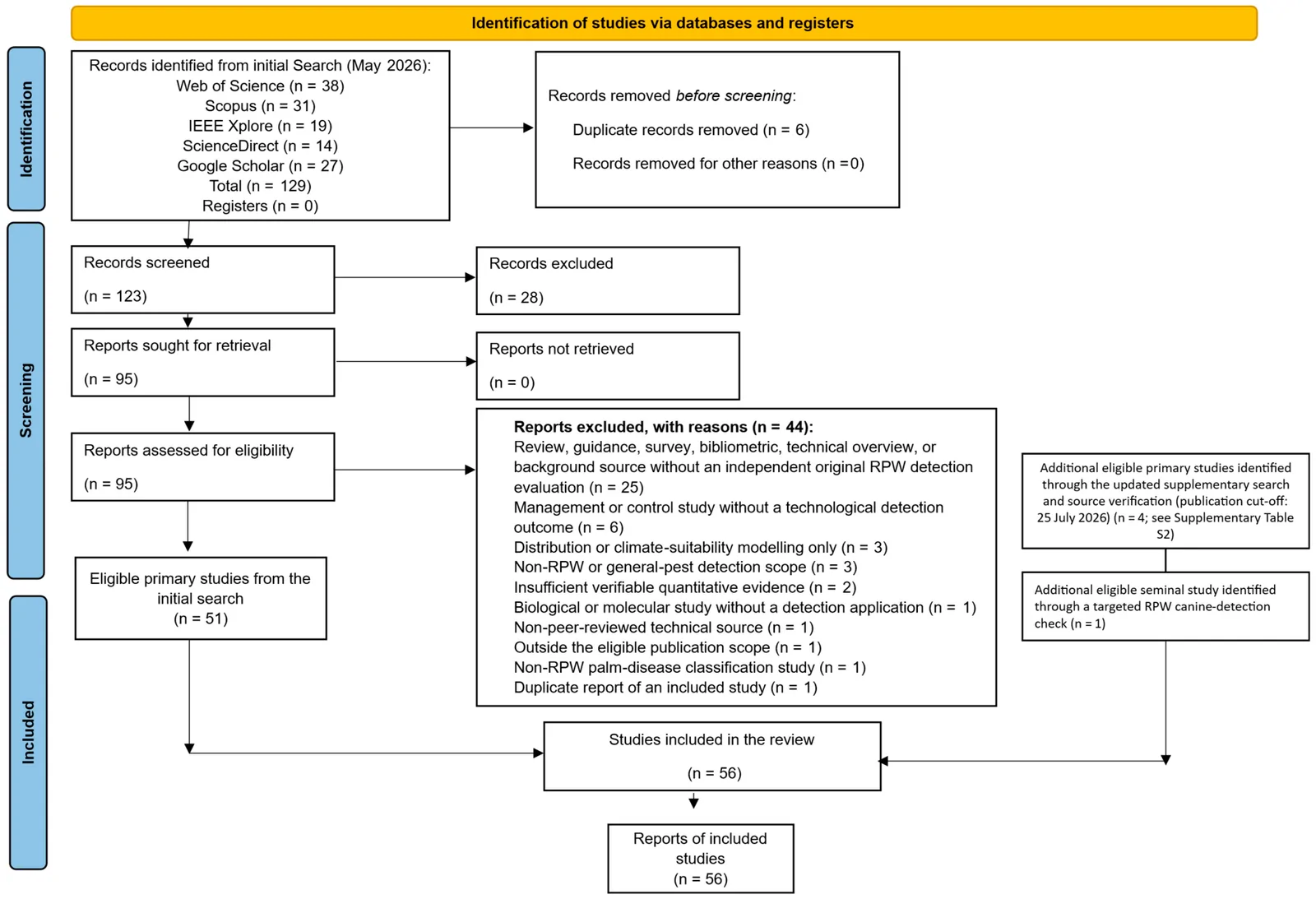
PRISMA 2020 flow diagram of the study identification, screening, eligibility assessment, and supplementary search process.

To strengthen the temporal currency of the evidence base through the stated publication cut-off of 25 July 2026, a structured supplementary update and source-verification procedure was undertaken following the principal May 2026 systematic search. Web of Science Core Collection, IEEE Xplore, ScienceDirect, and Google Scholar were used to identify potentially eligible records published or indexed after the principal search, while SpringerLink and PubMed supported publisher-platform and biomedical source verification. The predefined eligibility criteria were applied consistently, and records were checked against the existing corpus using titles, authors, and DOIs. This process identified four additional eligible primary studies. The supplementary procedure complemented the principal systematic search but was not designed as a complete rerun of every original database; Scopus was not separately rerun. The two procedures are therefore reported as distinct but complementary identification pathways in Figure 3 and Supplementary Table S2. A targeted supplementary check was subsequently conducted specifically for RPW-related canine detection. Because canine-specific terms were not represented as a dedicated search block in the original technology-focused strategy, this check was restricted to canine detection and did not constitute a rerun or general expansion of the systematic search. Full documentation is provided in Supplementary Table S2.

### 2.3. Eligibility Criteria and Study Selection

Eligibility criteria were defined before screening and are presented in Table 1. Studies were eligible when they investigated the detection, identification, or monitoring of *Rhynchophorus ferrugineus* using conventional, sensing, imaging, artificial-intelligence, or IoT-enabled approaches and reported original quantitative, experimental, analytical, or computational evidence. Eligible formats included peer-reviewed journal articles, indexed conference papers, and scholarly book chapters. Studies were required to report at least one assessable detection outcome.

Publications were excluded when they focused on non-RPW pests, management without a detection outcome, distribution or climate modelling, biological processes without a detection application, or insufficiently verifiable evidence. Non-English publications, editorials, abstracts, theses, and non-peer-reviewed reports were also excluded. Pre-2015 studies were retained only when they introduced or quantitatively validated a foundational detection technology.

Two reviewers (L.A.G. and A.A.) independently screened the titles and abstracts of all retrieved records and subsequently assessed the full texts of potentially eligible reports against the predefined eligibility criteria. Any disagreements were resolved through discussion and consensus. A formal inter-reviewer agreement statistic was not calculated. The initial search produced 129 records. After removal of six duplicates, 123 records underwent title and abstract screening, of which 28 were excluded. Ninety-five full-text reports were assessed, and 51 primary studies met the eligibility criteria. Four additional studies were identified through the supplementary update, and one additional eligible seminal study was identified through the targeted canine-detection check conducted during peer-review revision, yielding a final corpus of 56 primary studies (Figure 3).

### 2.4. Data Extraction, Methodological Appraisal, and Evidence Coding

A structured extraction framework was applied to all 56 studies. Nine variables were recorded: bibliographic information, detection-method category, algorithm or architecture, sensing modality and hardware, dataset characteristics, performance metrics, explainability method, targeted infestation stage, and validation environment. Elicit (https://elicit.com/, accessed on 30 September 2026) was used to support preliminary extraction, with all information manually verified against the original publications.

Methodological reporting and selected validation characteristics were appraised using a five-item checklist assessing:

1. Clarity and reproducibility of methodology.
2. Adequacy of dataset size and description.
3. Use of cross-validation or independent holdout evaluation.
4. Comparison with a baseline or state-of-the-art method.
5. Explicit reporting of limitations.

Studies scoring 4–5 points were classified as high, 2–3 as moderate, and 0–1 as low. Indicators that were absent, insufficiently reported, or unverifiable were scored as 0. The appraisal evaluated reporting completeness and selected aspects of validation; it was not intended as a formal risk-of-bias assessment. These categories describe fulfilment of the checklist criteria and should not be interpreted as validated ratings of methodological quality, risk of bias, or field readiness. No fixed modality-specific sample-size thresholds were applied; dataset adequacy (C2) was judged contextually according to the study design, number of independent biological or acquisition units, class distribution, acquisition setting, and completeness of dataset description. Study-level findings are reported in Supplementary Table S1. Operational scoring definitions for criteria C1–C5, including modality-specific considerations used in their interpretation, are provided in Supplementary Table S1.

Across the 56 studies, 40 received high appraisal scores, 15 received moderate scores, and one received a low score. Cross-validation or independent holdout evaluation was absent or insufficiently reported in 26 studies, explicit limitations were missing in 20, and dataset size or description was inadequate in 16.

Variables not consistently reported across the primary studies were classified using predefined review-level criteria. These included infestation stage, field validation, relative cost, scalability, automation, operational maturity, Technology Readiness Level, evidence strength, and multimodal fusion strategy. The classifications were assigned for comparative purposes and were not treated as directly reported study outcomes. Indicative TRL assignments were mapped to the conventional Technology Readiness Level framework using the following operational definitions: TRL 2, technology concept formulated; TRL 3, analytical or experimental proof-of-concept; TRL 4, component or subsystem validation in a laboratory environment; TRL 5, validation in a relevant environment; and TRL 6, prototype or system demonstration in a relevant environment [21]. Where the reviewed evidence spanned adjacent readiness levels, a range was reported rather than assigning an unsupported single value. TRL assignments reflected the demonstrated maturity of the RPW-specific approach rather than the maturity of individual commercial sensors or computing components. Coding was performed by the first reviewer and checked by the second. When evidence was insufficient to support a classification, the variable was recorded as not reported (NR).

### 2.5. Evidence Synthesis and Analysis

Evidence was synthesised using narrative analysis and comparative tables. A meta-analysis was not performed because the included studies differed substantially in sensing methods, biological targets, validation procedures, and reported performance metrics. No common effect measure was available for quantitative pooling. Missing information was recorded as not reported (NR) rather than inferred.

Appraisal scores were not used as exclusion criteria or as formal quantitative weights in the synthesis. The single study with a low appraisal score was retained because it met the predefined eligibility criteria; however, evidence from studies with weaker reporting or validation was interpreted cautiously and was not used alone to support strong conclusions regarding field readiness or generalisability. In Section 9 and Section 10, interpretation of the evidence considered the appraisal results together with biological relevance, validation environment, independence of sampling units, and specific methodological concerns such as data leakage and pseudo-replication identified in the reviewed studies.

During the preparation of this review, the authors used ChatGPT (OpenAI, GPT-5.6 Sol) and Claude (Anthropic, Sonnet 5.5) to assist with manuscript structuring, evidence organisation, consistency checking, and language refinement. The tools were not used autonomously for database searching, study eligibility decisions, data extraction, or methodological appraisal. All AI-assisted outputs were reviewed and verified by the authors against the original sources where applicable.

## 3. Conventional Detection and Surveillance Methods

Conventional RPW surveillance methods differ in biological target, spatial scale, and diagnostic role. Visual inspection assesses external symptoms at the palm level, whereas pheromone trapping monitors free-flying adults at orchard or area scale. Canine screening and direct trunk measurements provide targeted assessment of selected palms, while smart traps enhance the temporal resolution and automation of adult surveillance. Despite their continued operational relevance, these approaches generally offer limited capability for detecting concealed, pre-symptomatic infestation.

### 3.1. Visual Inspection

Visual inspection remains a frontline surveillance method in many RPW-affected regions. Trained inspectors examine the crown, trunk, and frond bases for external signs such as holes, exudates, fibrous frass, and deterioration of the crown [1]. Its main limitation is that visible symptoms often emerge only after substantial internal tunnelling and tissue damage have occurred [1,4]. Consequently, visual inspection is useful for identifying and prioritising symptomatic palms but is inadequate as a stand-alone method for concealed, pre-symptomatic infestation. Its use is therefore better suited to surveillance programmes that combine complementary detection approaches [1,22].

### 3.2. Pheromone Traps and Smart Surveillance

Aggregation pheromone traps use synthetic ferrugineol, commonly combined with food-based kairomones, to capture adult RPW and support population surveillance [8]. Capture rates are affected by trap location, lure source, bait condition, temperature, and other field conditions [23]. Higher catches have been reported for traps positioned in shaded, relatively moist locations near infested palms and at orchard edges, particularly under moderate temperatures [23]. The principal biological limitation is that pheromone trapping detects free-flying adults rather than concealed larvae within a particular palm. Trap catches provide information on local adult activity and seasonal population trends, but they do not identify which palms are infested or quantify internal damage [1,24]. Pheromone trapping should therefore be interpreted as an area-level surveillance and early-warning approach rather than a palm-level diagnostic method [1,8].

### 3.3. Canine Detection

Canine olfactory screening has been explored as a targeted method for detecting odours associated with RPW infestation [25]. Early work by Nakash et al. [25] provided initial evidence of technical feasibility, while Suma et al. [26] subsequently reported mean true-positive indications of approximately 78% and false-positive indications of approximately 12.6% in artificially baited Canary Island date palms. However, these experiments did not establish diagnostic sensitivity or specificity under naturally infested field conditions, and the minimum detectable infestation remains insufficiently defined. The peer-reviewed RPW-specific canine-detection evidence base therefore remains limited, and robust validation under naturally infested field conditions is still lacking. Canine detection is consequently more appropriately considered a targeted screening or verification approach than a validated large-scale early-detection method.

### 3.4. Physical Devices and Smart Trapping Systems

Targeted non-invasive or minimally invasive devices have been investigated for direct examination of selected palms. These include thermal cameras, Tree Radar Unit (TRU), resistograph-based measurements, and penetrometer or densitometer approaches [5,27]. Comparative field evaluations have reported variable performance across sensing methods and infestation conditions, while thermal measurements are particularly sensitive to solar loading [5]. Smart electronic traps extend conventional pheromone surveillance through automated recording. Aldryhim and Al Ayedh (2015) [28] used smart traps with three-hour recording intervals to characterise diel flight activity and reported capture performance comparable to that of conventional bucket traps. These systems improve the temporal resolution but do not alter the underlying biological target: free-flying adult weevils.

### 3.5. Comparative Summary

Table 2 compares conventional RPW surveillance and targeted assessment methods by operating principle, reported performance, infestation stage, and key limitations. These methods serve complementary rather than interchangeable roles. Visual inspection and physical devices assess individual palms, pheromone and smart traps monitor adult activity at the area level, and canine screening provides targeted palm-level assessment. Their combined use can therefore support broader surveillance across different biological stages and spatial scales.

### 3.6. Limitations of Conventional Detection Methods

Conventional RPW detection methods have three main limitations. First, early concealed infestation remains difficult to detect: pheromone traps indicate adult activity, whereas visual inspection and direct physical assessments often depend on symptoms or structural changes that appear after internal infestation has progressed. Second, per-palm inspection, repeated field visits, and reliance on trained personnel limit scalability in large plantations. Third, conventional observations are often not recorded in a standardised, time-resolved manner. Smart traps partly address this limitation by enabling time-resolved monitoring of adult captures, but they do not confirm internal infestation at the palm level. These limitations underpin RG-1 and RG-3 and motivate the acoustic and vibroacoustic approaches discussed in Section 4.

## 4. Acoustic and Vibroacoustic Detection Methods

### 4.1. Biological Signal and Sensor Taxonomy

Acoustic and vibroacoustic methods detect mechanical vibrations and sound generated by *Rhynchophorus ferrugineus* larvae during feeding and movement within palm tissues. These signals may support pre-symptomatic detection of active larvae, although detectability depends on larval activity and environmental conditions [4,29].

Three principal sensing configurations have been investigated. Contact vibroacoustic sensors, including piezoelectric probes and accelerometers, capture substrate-borne vibrations transmitted through palm tissue [29,30]. Microphone-based systems may use electret microphones in probe-coupled configurations [31] depending on the acquisition design. Distributed acoustic sensing based on phase-sensitive optical time-domain reflectometry (Φ-OTDR) measures strain-induced changes along an optical fibre and can extend monitoring across multiple palms from a single interrogation system [10,16,32]. These configurations differ in spatial coverage, sensor coupling, deployment complexity, and susceptibility to environmental interference.

### 4.2. Acquisition Hardware and Deployment Configurations

Contact sensors have been used to acquire trunk-borne larval activity [4]. Targeted orchard surveys have also employed contact-probe acoustic sensing [33]. Semi-autonomous monitoring nodes have been developed for continuous vibration-based monitoring [30]. In Saudi Arabian orchards, contact-probe recordings showed distinguishable temporal and spectral activity patterns for *R. ferrugineus* and *Oryctes elegans* under field noise, supporting the feasibility of species-oriented acoustic scouting [33]. Abioacoustic device detected activity from two-week-old RPW larvae in palms infested with five individuals by analysing sound intensity around 2250 Hz under controlled environmental conditions.

IoT-oriented platforms such as TreeVibes combine trunk-coupled sensing with networked data acquisition for extended monitoring of wood-boring activity [34]. Within the evidence reviewed here, independent RPW-specific orchard validation of this platform was not identified. Φ-OTDR systems extend spatial coverage by sensing vibration-induced optical changes along a fibre deployed across multiple monitoring points [16]. Collectively, the reviewed hardware ranges from manually applied probes to permanently installed sensing networks; study-specific configurations and validation settings are summarised in Table 3.

### 4.3. Signal Representation and Analytical Processing

Rule-based systems have used burst timing, impulse-train statistics, and spectral activity patterns to distinguish putative larval signals from background noise [4,33]. Other implementations have used threshold-based and frequency-domain features in acoustic recordings [29,35]. Supervised pipelines have used MFCC-based representations [36]. Combined CQCC, MFCC, and BFCC feature images have also been evaluated in deep-learning pipelines [37].

In DAS systems, Rayleigh backscatter traces are processed to obtain temporal and spectral representations [32]. These representations have been analysed using fully connected ANN and CNN models [10].

Study-specific signal representations and reported performance are summarised in Table 3, while the associated AI architectures are examined in Section 6.

**Table 3:** Comparative summary of acoustic and vibroacoustic RPW detection studies (2008–2026).

Ref.	Sensor	Acoustic Features	Model/Classifier	Dataset Characteristics	Reported Performance	F1-Score	Stage	Field Validation	Key Limitations
[4]	AED-2000 with SP-1L probe	Spectral profile matching and temporal burst analysis	Rule-based analysis (DAVIS/Raven)	Neonate-inoculated palm fronds; enclosed and open-environment recordings (Aruba)	Larval bursts detected in >80% of neonate-inoculated palm fronds	NR	Early-instar larvae	Partial	Burst detection was not equivalent to classifier accuracy; intermittent activity and environmental noise
[33]	Contact acoustic probe	Temporal and spectral burst patterns	Listener-assisted and rule-based pattern discrimination	Saudi orchard palms and offshoots (RPW and *Oryctes elegans*)	Quantitative classification accuracy NR	NR	Larval activity	Yes	No unified accuracy or F1-score; field noise and possible species confusion
[35]	Piezoelectric sensor with acoustic cone	Log-spectral feature vectors	Vector quantisation/Gaussian mixture models	Laboratory and field recordings	Detection ratio up to 98.9%	NR	Larval	Partial	Best performance obtained under controlled or low-noise conditions
[31]	Electret microphone with aluminium probe	Frequency-specific threshold indicator	Rule-based threshold detection	Controlled early-infestation experiments	90% detection accuracy	NR	Early larval infestation	Partial	Limited independent field validation; performance depended on specific probe and configuration
[34]	Piezoelectric IoT platform (TreeVibes)	Spectrogram-based representations	Cloud-based deep-learning pipeline	Long-term TreeVibes recordings of wood-boring activity	94% accuracy (Xception; random 20% holdout)	0.91 (infested); 0.96 (clean)	Wood-borer larval activity; RPW-specific stage NR	Partial	Independent RPW-specific orchard validation was not identified
[10]	Φ-OTDR fibre-optic distributed acoustic sensing	Temporal- and frequency-domain DAS representations	Fully connected ANN and CNN	Laboratory reconstruction using playback of sounds recorded from approximately 12-day-old larvae, with simulated wind and bird noise.	99.9% (ANN, spectral); 99.7% (CNN, temporal) for combined data with and without wind.	NR	Playback of early-larval sounds (~12-day-old larvae)	Partial	Primarily laboratory-based; open-air multi-site robustness not demonstrated
[16]	Φ-OTDR fibre-optic distributed acoustic sensing	Temporal and spectral DAS representations	Dual-branch CNN	18,000 infested and 18,000 healthy acoustic signal samples; training data derived from 6 artificially infested and 10 healthy trees; outdoor tests used 2 infested and 17 healthy trees	97.0–97.1% classification accuracy	NR	Early larval activity	Yes	Sensitivity to wind and environmental vibration; specialised fibre-optic infrastructure required
[38]	Acoustic recordings (acquisition hardware NR)	LPC spectral features with cosine-similarity matching	Bidirectional LSTM	≈3000 balanced RPW/non-RPW acoustic segments; independent palms/sessions/sites NR; controlled dataset.	98.02% classification accuracy, 93% test accuracy	97 as reported; scale unclear	Claimed early infestation; precise stage NR	No	Evaluation limited to controlled dataset; large-scale field validation lacking

### 4.4. Cross-Study Limitations and Operational Implications

Acoustic sensing remains constrained by the dependence of detectability on active larval movement and feeding. Intermittent activity can therefore produce periods in which infestation-related signals are weak or absent [4,29]. Environmental noise and wind can further degrade field recordings, particularly in contact-sensor and DAS deployments [16,33]. Signal transmission can also vary with recording geometry and the distance between larval activity and the sensor, limiting transferability across acquisition conditions [33]. Differentiation from other wood-boring insects remains relevant in orchards containing multiple pest species [33].

Reported outcomes are not directly comparable because the studies evaluated different tasks and units of analysis. Some reported burst-detection rates, others reported classification accuracy under controlled conditions, and others reported qualitative or field-based discrimination. Independent multi-site validation remained limited across the reviewed evidence, and DAS required more specialised infrastructure than individual-palm contact sensing. These limitations underpin research gaps RG-1 and RG-3 in Section 10. The resulting vibroacoustic representations provide inputs to the AI- and IoT-enabled detection systems examined in Section 6.

## 5. Thermal, RGB, UAV and Other Imaging-Based Approaches

### 5.1. Thermal Infrared Imaging

Thermal infrared (TIR) imaging detects physiological changes associated with RPW infestation by measuring temperature variation linked to altered tissue function and water transport [39,40]. Unlike acoustic sensing, which captures larval feeding activity directly, thermal imaging provides an indirect assessment of infestation-associated physiological stress rather than direct evidence of larval presence or activity. Measurements using data loggers inserted into drilled trunk holes showed higher internal temperatures in experimentally infested palms than in healthy palms.

Field evaluations have reported variable thermal discrimination performance depending on acquisition protocol, environmental conditions, and infestation severity. Pugliese et al. [5] compared thermal responses among neighbouring palms and considered measurements acquired under different solar-radiation conditions. Other single-site thermal studies have reported high classification scores, but often on limited, imbalanced, or non-externally validated datasets (Table 4). Thermal imaging remains sensitive to ambient temperature, solar loading, acquisition time, and viewing angle [40]. It is therefore best regarded as a complementary screening modality rather than a replacement for activity-based sensing.

### 5.2. RGB and UAV-Based Remote Sensing

UAV platforms enable non-destructive orchard-scale acquisition and, where repeated surveys are conducted, temporal monitoring of canopy and thermal changes. Delalieux et al. [12], in a two-year single-site proof-of-concept, reported thermal and spectral differences associated with severely infested palms, including observations made before clear canopy symptoms became externally visible. However, no validated diagnostic classifier, transferable diagnostic performance metric, or evidence of reliable early-stage detection was reported.

RGB imaging supports the automated detection of externally observable symptoms. Kagan et al. [11] combined aerial imagery for large-scale palm mapping with street-view imagery for classification based on visible crown symptoms, illustrating that RGB-based approaches are primarily suited to externally observable symptoms and are therefore more likely to represent established or externally manifested damage. Machine-learning and deep-learning methods applied to UAV and RGB imagery are reviewed comparatively in Section 6.

### 5.3. Microwave and Dielectric Sensing

Microwave sensing can penetrate vegetation and probe internal dielectric contrasts. Arroud et al. [14] demonstrated the detection and localisation of an RPW pupal phantom embedded within natural palm tissue under controlled conditions using a rotating antenna platform. Moradi and Bait-Suwailam [15] modelled the dielectric response of healthy and infested palm tissues and reported higher microwave reflection and lower absorption in infested tissue above 2.45 GHz. Both lines of evidence remain at analytical or controlled proof-of-concept stages; validation on naturally infested intact palms under plantation conditions is still required.

### 5.4. Cross-Modal Constraints and Operational Implications

Thermal contrast varies with ambient temperature, solar exposure, acquisition time, viewing angle, and infestation severity [5,40]. RGB and multispectral observations can be affected by solar geometry, atmospheric conditions, and canopy shadowing [12]. Such study-specific acquisition conditions constrain assessment of transferability across cultivars, sites, seasons, and hardware, underscoring the need for external validation [12,40] (Section 9.3).

Across the imaging and electromagnetic studies summarised in Table 4, three limitations constrain operational deployment. First, imaging approaches primarily capture physiological stress, tissue damage, or externally observable symptoms [11,12,40]. Microwave and dielectric approaches instead target internal electromagnetic contrasts but remain at proof-of-concept stage [14,15]. Second, geographic, sensor, and protocol heterogeneity, together with limited external validation, constrains assessment of cross-site generalisability [12,40]. Third, microwave and dielectric approaches remain predominantly at proof-of-concept stage [14,15]. These constraints are formalised in Section 10 as Research Gaps RG-1, RG-3, and RG-6.

The sensing modalities reviewed in Section 4 and Section 5 produce heterogeneous digital representations—acoustic spectrograms, thermal images, RGB imagery, and dielectric measurements. Section 6 reviews the machine-learning and deep-learning architectures that transform these outputs into detection decisions, and the IoT systems through which they may be integrated into connected monitoring architectures.

## 6. Artificial Intelligence, Machine Learning, and IoT-Enabled Detection

This section reviews representative artificial intelligence and machine-learning architectures across four biologically distinct task categories: (i) palm-level infestation detection or palm-health classification; (ii) adult-insect detection and recognition; (iii) RPW developmental-stage or specimen classification; and (iv) proxy or cross-species acoustic classification. These categories are not interchangeable. Palm-level infestation detection addresses whether an individual palm is infested, whereas adult-insect or specimen recognition identifies the organism outside the palm, and proxy tasks evaluate algorithmic behaviour on non-RPW-native data rather than RPW infestation diagnosis [37,44,45,46,47]. Because studies also differ in sensing modality, dataset provenance, and validation design, the synthesis is descriptive and does not constitute a direct benchmark ranking. Table 5 groups representative AI/ML studies by biological task category; Table 6 compares selected IoT-enabled systems.

### 6.1. Classical Machine-Learning Approaches

Classical machine-learning approaches have used tabular palm measurements, handcrafted image features, and thermal descriptors with classifiers such as random forests, support vector machines, shallow artificial neural networks, and ensemble methods [41,44,46,48,49]. Reported endpoints include adult-insect recognition, thermal palm-health classification, and tabular infestation prediction from temperature and morphology (Table 5). Conventional classical pipelines depend primarily on engineered or selected features, although hybrid approaches may combine learned deep representations with classical classifiers. This dependence has contributed to increased adoption of end-to-end representation learning.

### 6.2. Deep-Learning Architectures

Deep learning has become increasingly prominent in recent RPW-related automated analysis, applied to RGB images, thermal imagery, and acoustic spectrograms. Convolutional neural networks—including pretrained ResNet [47], DenseNet [37], EfficientNet [43], MobileNet [50], ShuffleNet [48], MixConvNet, and related families—are commonly adopted architectures. Lightweight networks and metaheuristic hyperparameter-optimisation procedures have been explored to improve efficiency and reported accuracy within individual datasets [43,48,50].

Reported performance must be interpreted strictly within each study’s biological target, dataset, and validation design. Adult-insect recognition, developmental-stage classification, broader palm-health classification, and proxy-borer acoustic classification should not be treated as equivalent evidence of concealed RPW infestation in an individual palm. Many models were trained on private, laboratory, single-site, web-sourced, or proxy corpora; direct cross-study accuracy comparison is therefore methodologically inappropriate (Section 8; Table 5). 

### 6.3. Explainability and Deployment-Efficiency Constraints

Recognised explainable-AI methods were rarely applied within the reviewed corpus. Martin et al. [42] used Grad-CAM qualitatively, but no quantitative evaluation of explanation faithfulness, stability, or decision utility was identified. Qualitative discussion of features or visual outputs does not constitute quantitative XAI evaluation. Given that RPW detection supports high-stakes phytosanitary decisions, this gap limits accountability and is formalised as Research Gap RG-4 in Section 10.

Lightweight model families, including MobileNet [50], ShuffleNet [48], and EfficientNet variants [43], have been examined alongside embedded computing platforms such as Raspberry Pi [42] and IoT sensing nodes [30].

TreeVibes illustrates networked acoustic acquisition [34] rather than, by itself, a standardised edge-inference benchmark. Evaluation remains dominated by classification scores; quantitative reporting of latency, memory, energy, and on-device throughput remains uncommon, constraining the objective comparison of deployment readiness (Table 5 and Table 6).

**Table 5:** Representative AI/ML studies by biological task category.

Study	AI Architecture	Input Modality	Task Category/Biological Target	Dataset	Reported Performance	F1	XAI	Deployment	Key Limitation
[45]	Faster R-CNN	RGB	Adult-insect detection and localisation	300 Google Image Search RPW images; augmented to 6000 images; 80/20 train–test split	99%	NR	NR	No	Custom image dataset; insect detection/localisation rather than concealed palm infestation; no external validation
[46]	ANN (GLCM + colour features)	RGB	Adult-insect recognition (RPW vs. ant)	913 insect images	92.22%	NR	NR	No	Single RPW- vs.-ant dataset; 10-fold cross-validation; no external field validation
[36]	AlexNet	Acoustic (MFCC)	Biological Target: RPW-native acoustic sound classification (RPW vs. NoRPW)	240 training samples reported; testing-set size inconsistently reported; RPW audio derived from ~10 larvae in a single laboratory tree model; independent palms/sessions/sites NR; laboratory evaluation	99.2% accuracy with all field noises; 100% with wind noise only	NR	NR	Laboratory prototype	Artificial laboratory tree model with ~10 larvae; field noises were mixed with RPW recordings; no orchard-scale field validation
[49]	Shallow ANN	RGB	Adult-insect recognition	319 RPW images and 93 other-insect images	99.5% RPW; 93.5% other insects.	NR	NR	No	Foundational pre-2015 approach; limited dataset
[50]	Mask R-CNN + MobileNetV2 + GTO	RGB	Adult-insect recognition/localisation	RPW image dataset; size/class composition NR	99.27% accuracy	NR	NR	No	Dataset size and train/validation/test split not reported; no external validation reported.
[37]	DenseNet121 and other tested CNNs	Acoustic (CQCC + TreeVibes)	RPW-native acoustic infestation classification with additional proxy/environmental benchmark evaluation	Al-Ahsa RPW: 1106 recordings (531 infested, 575 non-infested; independent palms NR) + TreeVibes + ESC-10 + ESC-50	Best reported accuracy: 100% across the evaluated configurations	RPW dataset: NR; ESC-10: 0.973; ESC-50: 0.997	NR	No	Independent palm count and split grouping for the RPW recordings NR; additional benchmarks include cross-species/proxy datasets.
[51]	EfficientNetV2S; DenseNet169	Acoustic	RPW acoustic classification	RPW/healthy palm audio; size NR	95.57% (EfficientNetV2S)	NR	NR	No	Dataset details limited; no external field validation
[47]	Modified ResNet-34	RGB (specimen stages)	Developmental-stage/specimen classification	483 web-sourced images	98.0%	0.98	NR	No	Web-sourced; unclear split; possible leakage; no field validation
[48]	ShuffleNet + BSA + XGBoost	RGB	Adult-insect recognition	Custom RPW dataset	99.43%	NR	NR	No	Hybrid pipeline; no recognised XAI method reported
[52]	SVM for RPW; CNN for leaf-spot/blight-spot classification	Thermal (RPW); normal/RGB images also used for other palm diseases	RPW thermal classification within a broader palm-disease framework	Thermal palm dataset	92.8% SVM accuracy for RPW	NR	NR	No	No field deployment; no recognised XAI method reported
[53]	VGGish (transfer learning)	Acoustic	Proxy/cross-species acoustic classification	TreeVibes	94.5% accuracy; recall 95%	0.94	NR	No	Benchmark dependence; proxy, not RPW-native diagnosis
[44]	SVM, RF, MLP, and other classical classifiers	Tabular (temperature + morphology)	Palm-level infestation detection	1200 Saudi palm records	93.08%	0.935	NR	No	Single-region; resampling; no external validation
[54]	Fine-tuned InceptionResNet-V2	Acoustic	Proxy/cross-species acoustic classification	TreeVibes: 2485 recordings (1754 clean, 731 infested), 35 folders; 26/9-folder train/test split; independent trees NR.	97.18% test accuracy; 10-fold CV mean 94.53 ± 1.69%	NR	NR	Conceptual/Prototype	Cross-species TreeVibes proxy; independent tree count NR; no RPW-native orchard validation.
[38]	LPC + cosine similarity + Bi-LSTM	Acoustic	RPW-native acoustic infestation classification	Approximately 3000 RPW and non-RPW acoustic segments; independent palms, acquisition sessions, and sites NR; controlled dataset	98.02% classification accuracy, 93% test accuracy	97 (as reported; scale unclear)	NR	No	Segment-level controlled evaluation; independent palms, sessions, sites, and split procedure insufficiently documented; no large-scale field validation
[55]	EfficientDet-D0 + CHHO + LSTM	RGB	RPW image-based object detection and classification	RPW detection database cited by the authors; image-dataset size and class composition NR	Reported average accuracy of 99.89% at 3000 epochs across 10 displayed test images	NR	NR	Conceptual/cloud-oriented	Dataset size, class composition, and train/test partition insufficiently reported; no external field validation or demonstrated cloud/IoT deployment

Note: Reported outcomes are not directly comparable because studies differ in task, modality, dataset, sampling unit, and validation design. Task category and deployment are review-assigned. NR indicates not reported or not verifiable. XAI = NR indicates that no recognised explainability method was identified. The 2024 conference study listed in Table S1 and Sayed et al. [47] both reported datasets of 483 images; overlap between these datasets could not be verified.

**Table 6:** Representative IoT and edge-enabled systems for RPW-related monitoring.

Study	Hardware	Communication	AI/Analytical Processing	Modality	Computing Pattern	Key Limitation
[30]	BMA400 accelerometer node + LoRa gateway	LoRa to gateway; IoT platform connectivity	Signal processing and statistical infestation fingerprinting; no validated AI classifier reported	Vibration	Hybrid architecture; edge AI not demonstrated	Proof-of-concept; limited quantitative classifier and deployment metrics
[54]	TreeVibes sensing platform	Wireless IoT/cloud transfer	Fine-tuned InceptionResNet-V2	Acoustic	Cloud	Proxy TreeVibes corpus; no independent RPW-native orchard validation
[34]	TreeVibes vibroacoustic recorder	Wireless cloud transmission	Cloud-based Xception benchmark	Vibroacoustic	Cloud	Cross-species wood-borer platform; not an independently validated RPW orchard diagnostic system
[56]	IoT acoustic acquisition + UAV imagery	Architecture incompletely reported	Separate acoustic classification, YOLOv8-based UAV palm detection, and geospatial mapping	Acoustic + UAV RGB	Not confirmed	Modalities processed in separate stages; fusion and computing location unclear
[42]	Raspberry Pi 4B + USB LWIR thermal camera	Local interface; network protocol NR	On-device quantised thermal CNN	Thermal	Edge	Controlled single-site configuration; no multi-farm external validation
[57]	Raspberry Pi 4 + ESP32-WROVER prototypes	Protocol NR	CNN on Raspberry Pi (99.02%); simple NN on ESP32 (98.79%)	Acoustic	Edge	Dataset, communication protocol, and field-deployment scope incompletely reported
[58]	Vibration-sensing nodes; implementation details NR	Data-mule concept; protocol NR	ResNet50, DenseNet121, Xception, and Inception-based vibration classification; performance NR	Vibration	Not confirmed	Full hardware, computing location, and evaluation details require source verification
[59,60]	Microphone sensor + stem-image acquisition	IoT communication details unclear	Multimodal deep-learning classification	Acoustic + RGB	Not confirmed	Possible overlap between COMICS and RED-BIYO records; edge/cloud architecture and validation details require reconciliation

### 6.4. IoT System Architectures for RPW Monitoring

Reviewed systems fall into three broad patterns. Cloud-based inference transmits sensor data to remote servers for processing (e.g., TreeVibes-centred frameworks). Edge-based inference executes models on embedded hardware; Martin et al. [42] provide one of the clearer reported edge demonstrations by deploying a thermal CNN on a Raspberry Pi 4B and reporting selected latency, model-size, and power characteristics under the tested configuration. Hybrid architectures combine local nodes, gateways, and cloud services for storage and analytics, but quantitative comparisons among cloud, edge, and hybrid designs remain scarce [30]. A solar-powered IoT system has been proposed to integrate remote palm monitoring, weevil tracking and trapping, and user alerts.

Communication architecture influences deployment coverage and data transfer, yet systematic comparisons under realistic orchard conditions remain limited (Table 6).

### 6.5. Environmental Context Integration

Environmental variables are widely acknowledged as sources of measurement variability [12,16], but their use as active inference inputs remains limited. No system identified within the reviewed corpus demonstrated prospectively validated environmental adaptation of thresholds, confidence estimates, signal-quality control, or acquisition scheduling. This gap motivates the framework discussed in Section 7 and Research Gap RG-6 in Section 10.

## 7. Multimodal Fusion and a Proposed Weather-Aware Framework

### 7.1. Current State of Multimodal Approaches

Within the reviewed evidence, no single sensing modality consistently satisfied the combined requirements for reliable early RPW detection across diverse field conditions. Acoustic and vibroacoustic sensing can capture signals associated with larval activity but remain susceptible to ambient noise, sensor–stem coupling, and wind-induced vibration. Thermal imaging detects infestation-associated physiological stress but is influenced by solar radiation and ambient conditions. RGB and UAV-based imaging primarily capture externally visible canopy symptoms that generally appear at more advanced stages. These complementary strengths and limitations motivate multimodal strategies that integrate heterogeneous information sources.

Within the reviewed corpus, seven studies used two or more sensing or information sources within a unified RPW detection or surveillance workflow (approximately 12.5% of the 56 included primary studies). These comprised explicit multimodal-fusion systems, multi-sensor temporal analysis, and sequential multi-source integration approaches, combining acoustic signals, RGB and thermal imagery, UAV and multispectral sensing, microwave backscatter, and geospatial information (Table 7). Most implementations integrate only two modalities; comprehensive combinations of acoustic, thermal, visual, and environmental sensing remain largely unexplored. Reported systems differ in sensing configuration, fusion strategy, deployment architecture, and validation design, preventing direct performance comparison. Current multimodal work should therefore be regarded as promising proof-of-concept rather than mature operational solutions for large-scale plantation monitoring.

COMICS and RED-BIYO share an author and report closely aligned acoustic–visual pipelines, including identical output classes, related preprocessing and notification components, and the same overall accuracy of 99.54%, although different model architectures are described. Because dataset and experimental independence could not be established, the publications were retained as distinct methodological reports but were not treated as independent validation evidence.

### 7.2. Proposed Six-Layer Weather-Aware AI Framework

**Figure 4:**
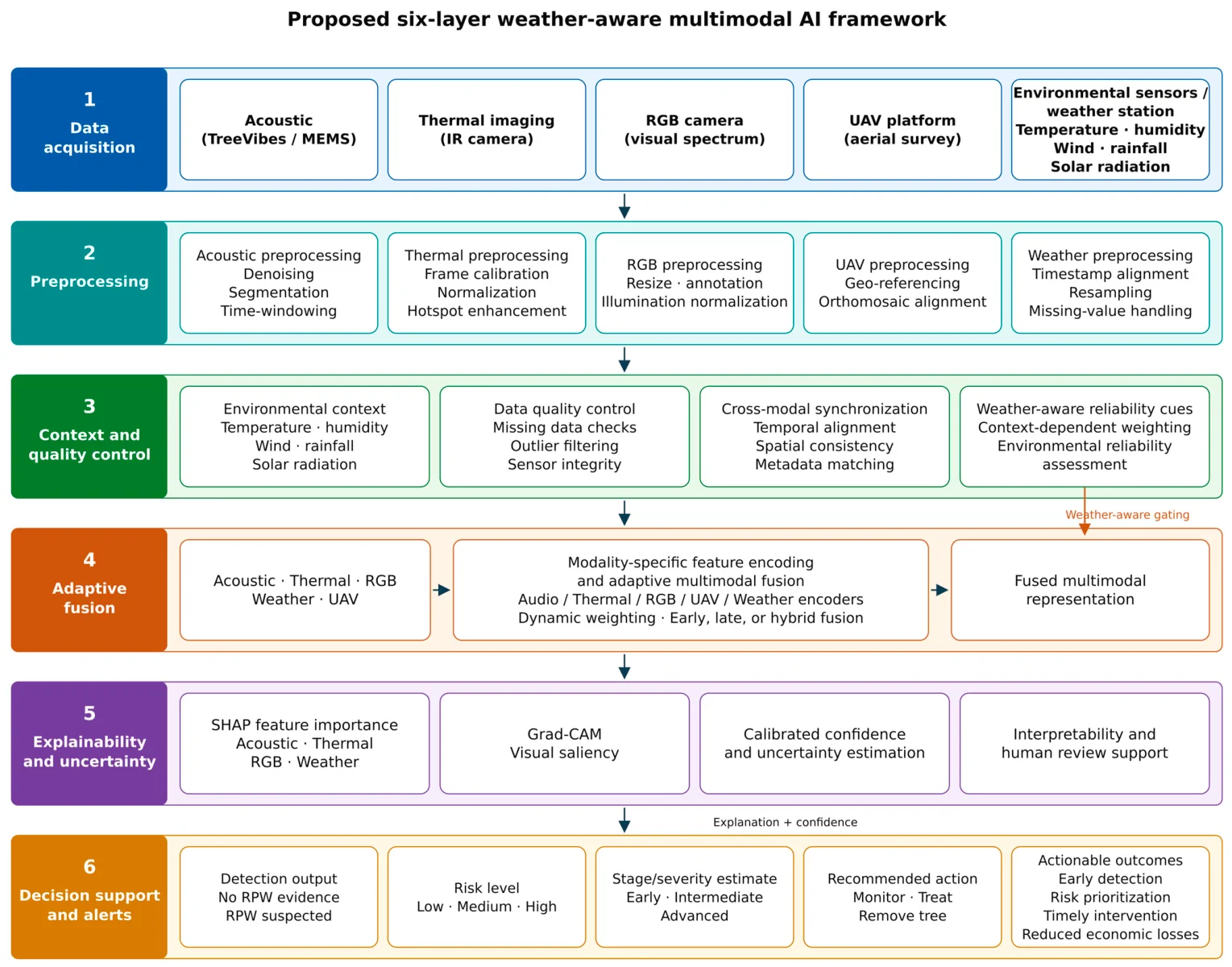
Proposed six-layer weather-aware multimodal AI framework for early RPW detection. The framework is conceptual and has not yet been experimentally validated as an integrated system. Numbers 1–6 denote the framework layers. Arrows indicate the proposed information flow, with the orange arrow representing weather-aware gating of multimodal fusion.

The systematic-review evidence synthesised in Section 4, Section 5, Section 6 and Section 7.1, is distinct from the conceptual framework presented below. Building on that evidence, this review proposes a six-layer weather-aware AI framework as a forward-looking reference architecture for future intelligent RPW monitoring systems (Figure 4). The functions assigned to these layers represent design requirements derived from the identified evidence gaps and should not be interpreted as capabilities already validated within the reviewed corpus. Accordingly, functions such as adaptive environmental weighting, uncertainty-aware rejection, and explainability are proposed research requirements rather than evidence-validated components of an existing RPW detection architecture.

- **Layer 1**—Multimodal Data Acquisition coordinates complementary sensors (acoustic, thermal, RGB, UAV, and environmental) according to the modalities available in a given deployment.
- **Layer 2**—Data Pre-processing applies modality-specific denoising, enhancement, normalisation, and temporal synchronisation where applicable.
- **Layer 3**—Environmental Context incorporates variables such as wind speed, ambient temperature, relative humidity, rainfall, and solar radiation to support context-aware quality control, reliability weighting, and adaptive inference, where empirically validated.
- **Layer 4**—Adaptive AI Fusion Engine combines available modality evidence using feature-level, decision-level, or hybrid fusion, accommodating variation in sensing reliability under changing field conditions.
- **Layer 5**—Explainability and Uncertainty provides modality- and feature-level attribution, uncertainty estimates, and abstention or referral mechanisms for low-confidence cases. These functions directly address the explainability and reliability gaps identified in Section 6.3.
- **Layer 6**—Decision Support and Alerts translates predictions into operational outputs through dashboards, mobile notifications, geospatial visualisation, and management guidance.

The six-layer framework is architecture-agnostic and can be implemented using the cloud, edge, or hybrid IoT patterns identified in Section 6.4. In a cloud-oriented implementation, sensing and initial acquisition occur in the field while computationally intensive preprocessing, fusion, inference, and decision-support functions may be performed remotely. In an edge-oriented implementation, a greater proportion of preprocessing and inference is executed locally on embedded or gateway hardware. Hybrid implementations can distribute these functions between field nodes, gateways, and cloud services according to connectivity, computational, and operational requirements. This mapping is conceptual and does not imply that any single reviewed system has validated all six layers as an integrated architecture.

Field implementation of the six-layer framework would also require explicit consideration of computational and energy constraints. RPW systems have demonstrated embedded or IoT-oriented processing, including Raspberry Pi-based thermal inference and networked sensing nodes [30,42], but systematic reporting of latency, memory, energy consumption, and on-device throughput remains limited. Accordingly, the proposed framework should not require all sensing and AI functions to operate continuously on a palm-mounted device. Computationally intensive fusion or model updating may be assigned to gateways or cloud services, while field nodes can prioritise sensing, data-quality checks, and lightweight or event-triggered inference where appropriate. Future implementations should therefore report processing latency, memory use, energy demand, communication load, and battery-life constraints alongside predictive performance to establish practical field readiness.

Collectively, the framework consolidates the principal findings of this review into an integrated conceptual architecture. Future work should implement and experimentally validate the complete pipeline under realistic plantation conditions and benchmark it against matched unimodal baselines.

## 8. Datasets, Benchmarks and Evaluation Limitations

### 8.1. Dataset Landscape

The reliability of RPW detection research depends on the quality, diversity, accessibility, and documentation of the datasets used for model development and evaluation. The reviewed literature reveals a fragmented landscape characterised by limited public availability and substantial dependence on institution- or project-specific corpora. Most acoustic, thermal, RGB, UAV, DAS, and microwave studies therefore cannot be independently reproduced or compared on identical data.

TreeVibes [34] is one of the few publicly accessible acoustic resources reused in RPW-oriented machine-learning studies, but its biological provenance requires explicit qualification. The original recordings were obtained primarily from mulberry trees affected by the cerambycid borer *Xylotrechus chinensis*, rather than from RPW-infested date palms. The resource should therefore be treated as a cross-species wood-borer proxy rather than an RPW-native benchmark. Moreover, reported model evaluations frequently used subsets of the full corpus, making the number of independent trees, sessions, and biological sources more informative than the raw number of extracted recordings or signal segments [37,54]. The biological transferability of TreeVibes to RPW should therefore be interpreted cautiously. Both *X. chinensis* and RPW generate substrate-borne vibrations through internal larval feeding and movement, providing a plausible generic wood-borer signal; however, this does not establish an RPW-specific acoustic signature. TreeVibes itself acknowledges that different borer species and host substrates may produce different spectral characteristics. Consequently, performance obtained from this corpus may reflect a combination of generic boring activity, insect-specific biomechanics, and host-substrate transmission properties, and should not be interpreted as evidence of RPW-specific generalisation without validation on RPW-infested palms [4,34]. Detailed scale and availability information are summarised in Table 8.

Public RPW imaging resources remain scarce. Nadeem et al. [40] reported a dataset of 832 RGB and thermal images collected from 179 date palms in Khairpur, Pakistan—a valuable but single-site resource. Table 8 summarises the principal datasets identified within the reviewed corpus.

### 8.2. Dataset Characteristics and Recurrent Limitations

RPW datasets differ substantially in modality, acquisition setting, biological representation, and annotation design. Acoustic corpora capture internal feeding or movement signals; thermal and RGB collections represent temperature variation or external appearance; DAS and microwave approaches generate specialised measurements that are not directly comparable with conventional audio or image data. Acquisition ranges from controlled laboratory experiments to field surveys of naturally infested palms. Binary healthy–infested labels predominate, while validated stage, severity, and developmental annotations remain uncommon.

Several recurrent limitations constrain reliability and generalisability (Table 9). Many datasets are small or based on limited numbers of independent palms, recordings, images, or signal segments. Large numbers of signal windows or image crops did not necessarily represent equally large numbers of independent palms, sites, or acquisition sessions, creating a risk of pseudo-replication. Geographic concentration was substantial across the reviewed corpus, limiting climate, cultivar, and population diversity. Environmental and sensor-position metadata are inconsistently documented. Class imbalance and sampling bias may inflate accuracy, and web-sourced images frequently over-represent advanced infestations. Finally, no dataset identified within the reviewed corpus provided temporally synchronised acoustic, thermal, and visual observations from the same palms. These constraints restrict early-stage detection, multimodal fusion, stage-aware modelling, and cross-site validation.

### 8.3. Benchmarking, Metrics, and Reproducibility

Dataset heterogeneity prevents direct model-to-model comparison: reported results arise from different modalities, class distributions, acquisition environments, annotation schemes, and experimental designs. Performance differences may therefore reflect dataset difficulty rather than algorithmic superiority. Random splitting of recordings, signal segments, frames, or image crops can place observations derived from the same palm, recording session, or site in both training and test sets, creating data leakage and optimistic estimates. Future evaluations should use palm-, session-, and site-grouped splitting, retain an untouched test set, and, where feasible, evaluate transfer across independent farms, cultivars, seasons, sensors, or regions (Table 9). Accuracy alone is insufficient under class imbalance, and precision–recall analysis may be more informative than ROC analysis when the positive class is rare [63,64]. For RPW applications, we therefore recommend reporting recall, false-negative rate, precision, false-alarm burden, specificity, ROC-AUC, PR-AUC, class prevalence, and confidence intervals. Operational evaluation should prioritise recall and false-negative rate while also reporting precision or false-alarm burden, because missed infestations permit continued spread whereas excessive false alerts increase inspection and intervention costs. Precision, F1-score, specificity, ROC-AUC, and PR-AUC should be reported jointly, together with class prevalence and confidence intervals.

Reproducibility remains limited because many studies use private datasets, incompletely describe preprocessing, and do not release code, model weights, or fixed partitions. Future work should follow FAIR principles [65] and, where ethical and legally permissible, provide openly accessible datasets, annotations, code, trained models, metadata, and fixed evaluation partitions. A shared, versioned RPW benchmark with fixed partitions and a transparent leaderboard would support more reliable comparison. Accordingly, the performance values discussed in Section 9 should be interpreted as dataset- and modality-specific rather than directly comparable.

## 9. Comparative Synthesis

This section synthesises evidence from Section 3, Section 4, Section 5, Section 6, Section 7 and Section 8 to compare RPW detection and surveillance approaches by operational purpose, detection capability, environmental robustness, automation, and technology maturity. Owing to heterogeneity in targets, datasets, sampling units, infestation stages, acquisition conditions, and evaluation protocols, Table 10 and Table 11 present descriptive comparisons rather than direct rankings. Table 10 compares the operational characteristics and reported evidence of the reviewed technologies, whereas Table 11 separately presents review-assigned operational maturity, indicative technology readiness, confidence, and the milestones required for further deployment.

### 9.1. Cross-Technology Performance and Readiness

The reviewed technologies address different operational tasks and biological endpoints. Visual inspection, canine screening, acoustic sensing, and thermal imaging are used primarily for palm-level assessment; pheromone traps monitor adult population activity; UAV systems support orchard-scale surveillance; and DAS, microwave sensing, and multimodal AI represent emerging architectures. Adult-insect recognition and developmental-stage classification identify specimens rather than diagnosing infestation within a palm. TreeVibes-based analyses often rely on a cross-species wood-borer corpus rather than an RPW-native date-palm benchmark. Reported outcomes therefore include surveillance observations, sensitivity estimates, signal classification, specimen recognition, and palm-level diagnostic decisions, which should not be treated as a single comparable endpoint.

A recurring trade-off exists between operational maturity and sensitivity to concealed infestation. Visual inspection is an established palm-level scouting practice, whereas pheromone trapping is an established population-surveillance method; neither provides direct information on early internal infestation. Contact acoustic sensing offers a biologically plausible pathway for detecting active larval feeding before external symptoms emerge, although sensitivity depends on infestation stage, sensor coupling, palm characteristics, and background noise. Networked vibroacoustic systems enable continuous monitoring, but results derived from multi-species corpora require cautious interpretation when generalised to RPW-only field deployment. Thermal imaging and UAV multisensor systems provide non-contact alternatives whose strongest evidence concerns established infestation or severe physiological and structural effects rather than biologically confirmed early infestation. DAS demonstrates high signal-classification performance under controlled and limited outdoor conditions, but evidence remains based on limited numbers of independent palms and experimental sites. Microwave sensing remains primarily analytical, while multimodal AI lacks synchronised acoustic, thermal, and RGB validation from the same palms. Table 11 summarises the corresponding operational-maturity and TRL judgments. Contact acoustic systems show greater practical maturity than most emerging sensing approaches, although robust multi-site validation remains limited. Thermal, UAV, and DAS systems occupy variable intermediate or prototype-level readiness, depending on the acquisition platform, automation level, and field evidence. Microwave sensing and multimodal fusion remain early-stage research technologies. High reported accuracy alone does not justify readiness advancement when evidence is derived from proxy species, small samples, controlled experiments, or non-independent test partitions.

### 9.2. Detection-Stage Coverage

The evidence does not support a universal week-based detection timeline because infestation progression varies with palm species, climate, larval number, infestation depth, and sensor configuration. A stage-oriented interpretation is therefore more appropriate.

**Figure 5:**
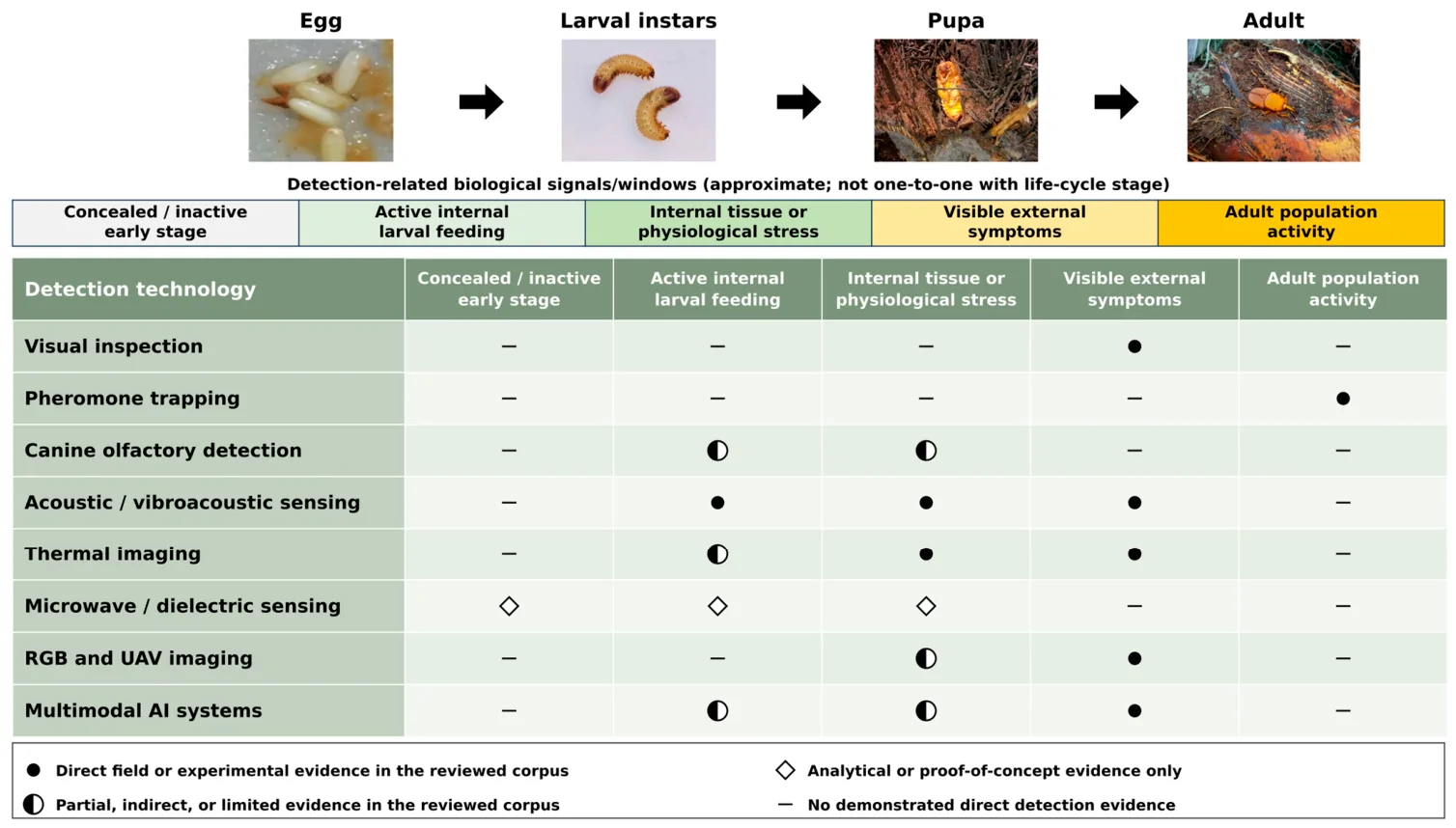
RPW life-cycle stages and stage-oriented detection windows across the reviewed technologies. Life-stage photographs adapted from [66,67] under CC BY 4.0. Note: Detection windows are approximate and stage-oriented rather than time-based. Symbols indicate evidence strength within the reviewed corpus; pheromone trapping reflects adult activity rather than infestation of an individual palm.

No reviewed field-deployable technology demonstrated validated direct detection of eggs or biologically inactive early infestation stages. Acoustic and DAS methods are most relevant during active larval feeding, although sensitivity is lower in the earliest phase. Thermal and spectral approaches become more informative when tissue damage, altered water status, or canopy stress generate measurable physiological changes. UAV evidence indicates that these changes may precede visible canopy symptoms, but current support is strongest for established or severe physiological and canopy effects. Visual inspection becomes more reliable only after external symptoms appear. The principal justification for multimodal monitoring is therefore stage complementarity rather than uniformly superior accuracy. Figure 5 summarises the stage-oriented evidence coverage of the reviewed technologies.

From an operational perspective, acoustic and UAV/spectral approaches therefore address different temporal and spatial roles. Contact acoustic and vibroacoustic sensing is primarily palm-level and is most informative when internal larvae are actively feeding or moving; it can provide evidence before external symptoms appear, but sensitivity may be reduced during very early or intermittent larval activity [4,29]. UAV-based RGB, multispectral, and thermal sensing provides broader spatial coverage and is better suited to screening multiple palms or plantation areas, but it predominantly measures host physiological or canopy responses rather than the insect directly [11,12]. Temporal UAV studies have demonstrated spectral or thermal differences before visible canopy symptoms, although the strongest evidence remains associated with established or severe physiological effects and requires further validation [12]. These characteristics suggest a complementary surveillance strategy in which wide-area UAV or spectral screening prioritises palms or zones for targeted palm-level acoustic assessment, rather than treating the two approaches as interchangeable diagnostic tools.

### 9.3. Environmental Robustness

Environmental conditions are a major source of domain shift. Acoustic and DAS systems are affected by wind, irrigation, machinery, wildlife, sensor coupling, and temperature-dependent larval activity. In one outdoor DAS experiment, performance deteriorated as wind speed increased, with more favourable results reported below approximately 9 mph under the tested configuration; this value should be treated as system- and site-specific rather than universal [16]. Thermal and UAV methods are influenced by solar loading, ambient temperature, humidity, shadows, viewing geometry, canopy structure, and acquisition time. Pheromone traps and canine screening are also environmentally sensitive through seasonal insect activity, lure condition, and odour dispersion. Future systems should evaluate whether weather and acquisition variables improve quality control, calibration, or conditional inference; any such adaptation should be prospectively validated.

Operational surveillance should also be adapted to the spatial and management context in which palms occur. In intensive plantations, structured trap networks can be combined with targeted palm-level sensing, whereas mixed or spatially heterogeneous agricultural settings may require selective, risk-based allocation of surveillance resources. Urban and ornamental landscapes impose different constraints because palms may be dispersed across public and private spaces; location-aware management systems and large-scale aerial/street-view mapping have demonstrated the value of spatially explicit surveillance in these environments [11,68]. Landscape-scale surveillance is also relevant because RPW adults tend to disperse over relatively short distances (<500 m) but have been recorded moving up to 7 km under field conditions, with dispersal increasing as temperature rises [69]. Palm-level diagnosis should therefore be interpreted alongside surrounding population surveillance rather than as an isolated activity. Seasonal and climatic conditions may further influence surveillance timing and spatial intensity, because pheromone-trap captures vary with temperature and local placement conditions, including proximity to infested palms, shade, and orchard edges [23].

### 9.4. Overall Synthesis and Evidence Boundaries

Promising predictive performance has been demonstrated under study-specific conditions, but operational translation remains constrained by limited RPW-native datasets, inadequate external validation, inconsistent environmental documentation, and the absence of synchronised multimodal benchmarks. The detailed limitations in Table 10 and Table 11 are formalised in Section 10 as research gaps and development priorities.

These findings should also be interpreted in light of limitations affecting both the underlying evidence and the review process. The included studies were heterogeneous in biological targets, sensing modalities, sampling units, ground-truth procedures, validation designs, and outcome definitions; therefore, direct performance ranking and quantitative meta-analysis were not appropriate. Many studies relied on small, private, single-site, proxy, artificially infested, or controlled datasets, limiting external generalisability. At the review level, the protocol was developed before the search but was not prospectively registered; a retrospective, time-stamped protocol record was subsequently deposited on OSF [20].

Eligibility was restricted to English-language publications with accessible full texts. This language restriction may have excluded relevant RPW studies published in Arabic or other non-English regional outlets, particularly from regions where RPW research is concentrated. Google Scholar and publisher-platform searches are less reproducible because rankings, indexing coverage, and query interfaces can change over time. The evidence base was established through a systematic multi-source search conducted in May 2026 and was further strengthened by a structured supplementary update and source-verification procedure through 25 July 2026. The supplementary procedure complemented the principal search but did not constitute a complete rerun of every original database; Scopus was not separately rerun. Consequently, the possibility that some recently published or newly indexed eligible reports outside the supplementary routes were not captured cannot be excluded. This limitation concerns the temporal coverage of the supplementary update and should be considered when interpreting the review’s synthesis and conclusion.

In addition, the review-specific appraisal assessed reporting completeness and selected validation characteristics rather than formal risk of bias. These limitations warrant cautious interpretation of cross-study comparisons, field-readiness claims, and technology-readiness judgments.

## 10. Research Gaps and Future Directions

The synthesis identifies eight research priorities that currently limit the transition of RPW detection from experimental feasibility to reliable operational deployment. Rather than repeating the dataset, performance, and maturity limitations discussed in Section 8 and Section 9, this section translates them into focused scientific and engineering directions. Table 12 summarises the evidence base, operational implications, and priority actions associated with each gap.

### 10.1. Reliable Early-Stage Detection

No reviewed field-deployable technology demonstrated validated direct detection of eggs or biologically inactive early infestation stages. Future evaluation should prioritise naturally infested palms with entomologist-verified infestation stage and independent ground truth. Where artificial inoculation is required, inoculation date alone should not be treated as sufficient evidence of the biological stage present at acquisition.

### 10.2. RPW-Native Benchmark Infrastructure

Available datasets remain fragmented, geographically restricted, and in some cases cross-species. A multi-site, multi-season RPW benchmark designed in accordance with FAIR principles [65] is needed. For RPW research, we propose that this benchmark include persistent palm identifiers linking co-located acoustic, thermal, RGB, environmental, and biological records; versioned public development partitions; reproducible public baselines; and, where sufficient scale and governance are available, an independently administered hidden test set.

### 10.3. Prospective Multi-Site Validation

Much of the current evidence derives from controlled experiments, artificial infestation, single sites, or retrospective analyses. Data partitioning should be applied at the palm, session, farm, or site level to prevent leakage. External test performance across farms, cultivars, climates, seasons, and sensor installations should precede strong claims of geographic generalisability or advancement to higher technology-readiness categories.

### 10.4. Explainability and Decision Accountability

Within the reviewed corpus, recognised explainable-AI methods were rarely applied, and no study was identified that quantitatively evaluated explanation faithfulness, stability, or decision utility, despite the phytosanitary and economic consequences of false alarms and missed infestations. Future systems should provide modality-specific explanations and evaluate their faithfulness, stability, and decision utility, while decision interfaces should distinguish model evidence, predictive uncertainty, and recommended field action.

Future RPW studies should match the explanation method to the input modality and model architecture. For CNN-based RGB, thermal, or multispectral imagery, Gradient-weighted Class Activation Mapping (Grad-CAM) could be used to highlight image regions that contribute most strongly to a model prediction [70]. For models based on structured predictors, such as spectral bands, vegetation indices, environmental variables, or engineered acoustic features, SHapley Additive exPlanations (SHAP) could be used to quantify feature contributions for individual predictions [71]. Local Interpretable Model-agnostic Explanations (LIME) could additionally provide local, model-agnostic explanations of individual predictions across different classifier types [72]. For CNN-based acoustic spectrograms, Grad-CAM could also be investigated as a means of identifying influential time–frequency regions; however, this represents a proposed application rather than an RPW-specific validation of the method. These explanation techniques should not be treated as evidence of model validity by themselves; future studies should also evaluate explanation faithfulness, stability, and usefulness for field decision-making.

### 10.5. Experimentally Validated Multimodal Fusion

Existing fusion studies frequently rely on independently acquired modality datasets, loosely aligned observations, or decision-level combination rather than temporally synchronised measurements from the same palms. Early, late, and adaptive fusion architectures should be compared against matched unimodal baselines trained and evaluated on identical partitions from the same palms, with modality-ablation, missing-modality robustness, calibration analysis, and external validation.

### 10.6. Weather-Aware and Domain-Adaptive Inference

Environmental variables are often treated as post hoc limitations rather than operational inputs. Thresholds derived from a single orchard or sensor configuration cannot be assumed to transfer across sites, seasons, or hardware. Validation should span multiple acquisition periods, cultivars, sensor installations, and locations, with explicit monitoring of calibration drift. Any environmentally conditioned, recalibrated, or domain-adaptive mechanism should be prospectively tested.

### 10.7. Edge-AI and Resource-Efficiency Benchmarking

Edge suitability is frequently claimed without systematic embedded-hardware evaluation. Complete sensing pipelines—including acquisition, preprocessing, inference, and communication—should be benchmarked on representative platforms for latency, energy, memory, throughput, communication load, and accuracy after compression or quantisation.

### 10.8. Stage- and Severity-Aware Diagnosis

Binary healthy–infested labels dominate, while validated progression and severity annotations remain uncommon. Severity labels should integrate converging evidence from infestation stage, larval burden, tissue damage, acoustic activity, thermal anomalies, and external symptoms. Longitudinal datasets tracking the same palms across sessions would support ordinal severity prediction and treatment-response analysis. Repeated temporal acquisition in prior field work illustrates the value of such designs, although validated severity modelling remains limited [12].

Collectively, these priorities should be evaluated not only through higher classification scores, but through validated early-stage detection, calibrated uncertainty, external generalisability, operational feasibility, and measurable improvement in phytosanitary surveillance and intervention decisions.

These research gaps also provide an evidence-based rationale for the conceptual six-layer framework introduced in Section 7.2. Layer 1 is primarily associated with RG-1 and RG-8; Layer 2 with RG-2 and RG-5; Layer 3 with RG-6; Layer 4 with RG-5 and RG-6; Layer 5 with RG-4; and Layer 6 with RG-3. RG-7 spans multiple layers because resource-efficient edge implementation affects sensing, inference, fusion, and deployment rather than a single architectural component. These associations are intended to show the main relationships between the framework layers and the identified research gaps; they should not be interpreted as exclusive one-to-one correspondences.

## 11. Conclusions

This systematic review synthesised 56 primary studies, primarily published between 2015 and July 2026, together with selected seminal pre-2015 contributions, to evaluate the principal technologies used for the early detection and surveillance of red palm weevil infestation. The reviewed evidence covered conventional inspection and trapping, acoustic and vibroacoustic sensing, thermal and optical imaging, UAV-based monitoring, artificial intelligence, IoT-enabled systems, distributed acoustic sensing, microwave approaches, and multimodal architectures.

The evidence demonstrates substantial progress in automated RPW monitoring, particularly in acoustic signal classification, thermal-image analysis, and remote sensing. However, high reported performance was frequently obtained under study-specific conditions involving small datasets, controlled experiments, artificial infestation, proxy species, or non-standardised evaluation procedures. Consequently, reported accuracy values should not be interpreted as directly comparable indicators of field effectiveness. Mature surveillance practices, including visual inspection and pheromone trapping, remain operationally scalable but provide limited information about concealed infestation in individual palms. Emerging technologies offer greater potential for internal or pre-symptomatic detection, although their readiness is constrained by limited external validation, environmental sensitivity, and uncertain deployment costs.

The review identifies dataset provenance, biological ground truth, environmental variability, and reproducibility as central determinants of model reliability. Existing datasets remain fragmented across modalities and locations, and no benchmark identified in the review provides synchronised acoustic, thermal, visual, and environmental observations from the same naturally infested palms. No included study explicitly implemented and quantitatively evaluated a recognised XAI method, limiting the transparency, interpretability, and accountability of model-supported decisions.

The principal contribution of this review is therefore not a ranking of detection technologies, but an integrated assessment of their operational purpose, biological coverage, environmental robustness, and technology readiness. It also consolidates eight research priorities involving reliable early-stage detection, RPW-native benchmark infrastructure, prospective multi-site validation, explainability, synchronised multimodal fusion, weather-aware inference, edge-AI benchmarking, and stage-aware diagnosis.

Future progress should be evaluated through biologically verified early detection, calibrated uncertainty, external field performance, reproducibility, and operational utility rather than classification accuracy alone. Addressing these priorities could support the development of more reliable, explainable, and environmentally robust RPW surveillance systems for precision palm protection.

## Figures and Tables

**Table 1:** Predefined inclusion and exclusion criteria applied during PRISMA screening (IC1–IC6/EC1–EC6).

#	Inclusion Criteria	Exclusion Criteria
IC1/EC1	Studies investigating detection, identification, or monitoring of RPW (*Rhynchophorus ferrugineus*)	Studies focused on pest species other than *Rhynchophorus ferrugineus*
IC2/EC2	Peer-reviewed journal articles, indexed conference papers, and scholarly book chapters reporting original experimental or quantitative evidence on RPW detection, identification, or monitoring.	Editorials, letters, opinion articles, abstracts, theses, non-peer-reviewed technical reports, and conference papers or book chapters that do not report original experimental data.
IC3/EC3	Studies published between 2015 and 25 July 2026, together with selected pre-2015 seminal studies that introduced or quantitatively validated foundational technologies for RPW detection.	Pre-2015 publications that did not introduce or quantitatively validate a foundational RPW detection method, and studies published after the 25 July 2026 search cut-off.
IC4/EC4	Studies employing a relevant RPW detection or surveillance technology—including acoustic/vibroacoustic sensing, computer vision, thermal or hyperspectral imaging, X-ray radiography, microwave or dielectric sensing, ML/DL, IoT-based sensing, biohybrid sensing, or quantitatively evaluated conventional monitoring—and reporting an assessable detection outcome.	Studies without an assessable RPW detection or surveillance outcome.
IC5/EC5	Studies reporting at least one verifiable outcome, including diagnostic performance metrics, detection rates, statistical separation, controlled comparative evidence, or experimentally demonstrated detection or localisation.	Duplicate publications or studies with overlapping datasets without a novel methodological contribution
IC6/EC6	Studies written in English with full text accessible	Non-English language publications or studies with inaccessible full text

**Table 2:** Comparative summary of conventional and field-oriented RPW detection methods.

Detection Method	Operating Principle	Reported Performance	Infestation Stage	Key Limitations	References
Visual Inspection	Expert inspection of crown, trunk, and frond bases for holes, exudate, frass, or wilting	Operator-dependent; low early-stage sensitivity	Established infestation; visible symptoms	Late detection after substantial damage; labour-intensive; low scalability	[1,22]
Pheromone Traps	Ferrugineol and food-kairomone traps for adult capture	Variable catch rates; useful for population surveillance	Adult stage only	Does not confirm larval infestation in an individual palm; placement- and maintenance-dependent	[8,24]
Canine Detection	Screening for RPW-associated odours	Approximately 78% true-positive indications and 12.6% false-positive indications in experimentally baited Canary Island date palms; diagnostic sensitivity and specificity under naturally infested field conditions remain unestablished.	Concealed adults or larvae in experimentally baited palms; natural early-infestation performance not established	Limited RPW-specific evidence; artificial-baiting validation; handler dependence, fatigue, training and operational cost; no large blinded multi-site validation on naturally infested palms	[25,26]
Densitometer	Probe-based assessment of changes in trunk density	Accuracy: 88.89% in Italy and 82.26% in Saudi Arabia; the highest Saudi ground-level accuracy was 87.10%	Infestation stage NR	Direct-contact, minimally invasive measurement; performance varied according to measurement position.	[5]

**Table 4:** Comparative summary of imaging and emerging sensing studies for RPW detection (2015–July 2026).

Study	Sensing Modality	Features/Method	Model/Approach	Dataset	Reported Performance	F1	Stage	Field Validation	Key Limitation
[39]	Thermal infrared imaging; handheld and/or aerial platform as reported	Canopy thermography; temperature contrast	Threshold-based interpretation	Multi-year controlled experiments and aerial thermal imaging in a commercial date-palm orchard, Israel	6 of 8 known-infested palms detected in one trial (75% sensitivity estimate); overall diagnostic accuracy NR	NR	Established infestation; stronger discrimination at later stages	Yes	Limited specificity and denominator reporting; trial-specific outcome; over-estimation reported
[12]	UAV RGB, multispectral, and thermal imaging	Crown segmentation; maximum temperature, thermal variability, and temporal vegetation indices	Segmentation and temporal analysis; no validated diagnostic classifier	18 palms (9 infested, 9 controls); two-year single-site study, Spain	NR	NR	Severe infestation; pre-visual thermal/spectral differences reported, but validated early-stage detection not demonstrated	Partial	Small single-site proof-of-concept; no transferable classifier or external validation
[40]	Paired RGB and thermal imaging	Thermal-histogram features; four health-status classes	Feed-forward neural network (20 neurons)	832 RGB/thermal images from 179 palms; 416 thermal images evaluated	63.7% overall classification accuracy across all 416 thermal images (combined training, validation, and test data)	NR	Health-status classes; biological infestation stage NR	Partial	Single-region dataset; labels based on visual inspection and farmers’ feedback; no external validation
[41]	Thermal imaging	K-means segmentation; shape, temperature, LBP, thermal-gradient, and colour-histogram features; SIFT and ORB feature matching	CNN, SVM, and RNN classifiers	Mixed web-sourced and directly acquired thermal images; total sample size and class distribution NR	RNN: 98.53% test accuracy and 98.97% validation accuracy	CNN: 0.9853; SVM: 0.875; RNN: 0.984	Claimed early detection; biological infestation stage NR	No	Mixed web/live-source images; total dataset size, class balance, and split procedure unclear; no external field validation
[42]	LWIR thermal imaging	Real-time thermal classification	IoT-edge CNN	6000 thermal images (class distribution inconsistently reported); independent palms/sessions NR; single-grove dataset.	98.5% image-level classification accuracy	0.97	Infestation present; precise biological stage NR	Partial	Single-grove dataset; inconsistent class-distribution, split, and metric reporting; random image-level split may permit palm/session leakage; independent palm count NR.
[11]	Aerial and street-view RGB imaging	Palm localisation and visible-symptom analysis	Faster R-CNN for palm detection; XResNet for symptom classification	47,138 aerial + 61,009 street-level palm images; independent palm count NR.	Palm mapping and visible-symptom detection demonstrated; unified RPW diagnostic performance NR	NR	Visible crown symptoms; biological infestation stage NR	Yes	Image counts do not represent independent palms; asynchronous aerial/street-view acquisition; weak biological ground truth.
[43]	RGB imagery	Transfer learning and few-shot recognition	ResNet50 or EfficientNet-B0 with MAML or prototypical networks	Approximately 293 images; 70/15/15 train/validation/test split	91.3% best reported classification accuracy	0.90 (EfficientNet-B0 + MAML)	RPW image recognition; biological infestation stage NR	No	Very small dataset; external few-shot generalisation not established
[14]	Microwave imaging with rotating Vivaldi antenna platform	S-parameter/backscatter measurements; DAS and DMAS image reconstruction	Radar-based microwave imaging with DMAS localisation	RPW pupal phantoms tested in three cut palm-trunk segments from different trees under controlled laboratory conditions	≈93% localisation accuracy in the experimental setup; diagnostic classification accuracy NR	NR	Pupal phantom; no natural biological stage evaluated	No	Controlled phantom study; no naturally infested live-palm or orchard validation
[15]	Microwave dielectric sensing simulation	Frequency-dependent dielectric response; reflection and absorption	Empirical dielectric modelling; reflection-to-absorption ratio (R)	Modelled healthy and infested palm tissue slabs	Higher reflection and lower absorption in infested tissue above 2.45 GHz; diagnostic accuracy NR	NR	Biological infestation stage NR	No	Theoretical/simulation study only; no hardware, biological, or field validation

**Table 7:** Multimodal and multi-source RPW detection and surveillance studies.

Study	Modalities	Integration Category and Strategy	AI/Analytical Model	Field Validation	Main Contribution
[56]	Acoustic (IoT) + UAV + geospatial	Sequential multi-source integration	Custom DL + YOLOv8	Partial	Sequential workflow combining IoT acoustic monitoring, UAV palm detection, and GIS mapping
[59] *	Acoustic + RGB stem images	Explicit decision-level fusion using fuzzy rules	CAB-RetinaNet (images) + PEN-BiGRU (audio)	Partial	Acoustic–visual multimodal fusion with IoT-enabled mobile alerts; computing location requires verification
[60] *	Acoustic + RGB stem images	Explicit decision-level fusion using fuzzy inference	YOLOv9 + SCNN-BiGRU	Partial	Acoustic–visual decision fusion with IoT-enabled farmer notification; edge deployment not established
[12]	UAV RGB + multispectral + thermal	Multi-sensor temporal analysis; no unified AI-fusion model	Vegetation-index/temporal analysis	Partial	Two-year UAV proof-of-concept using complementary sensing modalities
[11]	Aerial + street-view RGB	Sequential multi-source integration	Faster R-CNN + XResNet	Yes	Large-scale urban mapping of palms exhibiting visible symptoms
[61]	Acoustic + microwave backscatter	Explicit decision-level fusion using adaptive sequential Kalman filtering	Modality-specific classifiers + adaptive sequential Kalman fusion	Partial	Acoustic–microwave fusion achieved 96.2% reported accuracy on real-world datasets; sampling unit and multi-site external validation were not established
[62]	Paired RGB + thermal	Hybrid feature-level fusion with ensemble decision integration	ResNet-50, EfficientNet-B0, ConvNeXt-Tiny, Xception, ViT-Small, and ConvNeXt V2-Tiny	Partial	Four-class palm-health classification; ensemble accuracy 76.2% and macro-F1 0.756 under a tree-level split; fusion gains were modest and architecture-dependent; no multi-site external validation

Note. Integration categories and field-validation status are review-assigned. Partial indicates field-derived data or limited/single-site evaluation without independent operational validation. Outcomes are not directly comparable because tasks, fusion strategies, sampling units, and validation designs differ. * Sundari et al. [59] and Siron Anita Susan et al. [60] share an author and report closely aligned COMICS/RED-BIYO acoustic–visual pipelines. Dataset and experimental independence could not be established; therefore, they were retained as distinct methodological reports but not treated as independent validation evidence.

**Table 8:** Principal datasets and benchmark corpora used in RPW detection research (2015–2026).

Study/Corpus	Modality	Dataset Scale	Acquisition Context	Availability	Target/Labels	Principal Limitation
[34]	Vibroacoustic	57,841 recordings (4165 field; 53,676 laboratory); folders correspond roughly to individual trees.	Mulberry trees (Crete, Spain); primarily *Xylotrechus chinensis*	Public	Clean/infested borer activity	Large recording count reflects repeated recordings per tree; cross-species proxy, not RPW-native.
[36]	Acoustic (MFCC)	RPW audio derived from ~10 larvae in a single laboratory tree model; training/testing sample counts reported inconsistently.	Controlled laboratory; location NR	Not public	Healthy/infested	No independent palms; laboratory model with mixed field noise.
[32]	Fibre-optic DAS (Φ-OTDR)	Controlled recordings from two palms	Laboratory, Al-Hassa, Saudi Arabia	Not public	Healthy/infested	Very small biological sample; limited field generalisation
[10]	Fibre-optic DAS	Laboratory + reconstructed farm-noise recordings; size NR	Laboratory/simulated field noise, Saudi Arabia	Not public	Healthy/infested	No fixed partitions; limited external validation
[16]	Fibre-optic DAS + CNN	Controlled + outdoor field recordings; reusable size NR	Date-palm farm, Saudi Arabia	Not public	Healthy/infested	Single-site; data and partitions unavailable
[30]	Accelerometer/IoT	Study-specific; count NR	Laboratory and farm, Saudi Arabia	Not public	Infestation monitoring	No public annotations or fixed splits
[39]	Thermal infrared	Multi-year controlled and commercial-orchard thermal observations; exact dataset size NR	Israel	Not public	Healthy/infested	Single-region; limited annotation metadata
[40]	Paired RGB + thermal	832 images from 179 palms (416 thermal used)	Date-palm groves, Khairpur, Pakistan	Public (Figshare) *	Non-infected/infected/badly damaged/dead	RPW-native but single-site; no acoustic data
[42]	Thermal infrared	6000 images (class distribution inconsistently reported); independent palms/sessions NR.	Coconut grove, Tamil Nadu, India	On request	RPW/non-RPW	Single grove; inconsistent class-distribution reporting; image-level splitting; independent palm count NR.
[45]	RGB object detection	300 web-sourced images (pre-augmentation)	Online sources	Not public	Adult RPW detection	Web-scraping bias; does not represent concealed infestation
[46]	RGB classification	913 images (448 RPW + 465 ants)	Multiple sources	Not public	RPW versus ant	Adult-insect recognition, not palm-level infestation
[37]	Acoustic (RPW + proxy benchmarks)	Al-Ahsa RPW: 1106 recordings; independent palms/sessions NR + TreeVibes + ESC-10 + ESC-50	Existing public benchmarks	Mixed/partly public	RPW infested/non-infested + proxy/environmental classes	Derived benchmark; inherits TreeVibes species mismatch
[12]	UAV RGB + multispectral + thermal	18 palms, repeated over two years	Experimental plot, Spain	Not public as reusable corpus	9 infested + 9 control	Small controlled trial; advanced-stage focus
[11]	Aerial + street-view RGB	47,138 aerial + 61,009 street-level palm images; independent palm count NR.	Online urban imagery (mainly Israel)	Source images online; curated labels not public	Palm detection + suspected infestation	Multiple images may represent the same palms; asynchronous viewpoints; limited biological ground truth.

Note. DAS = distributed acoustic sensing; NR = not reported. “RPW-native” denotes data from verified RPW specimens or RPW-infested palms. TreeVibes is retained because it has been reused in RPW-oriented studies, but its original corpus is principally associated with *X. chinensis* in mulberry and should be labelled a cross-species proxy. ESC-50 is a general environmental-sound corpus and should not be counted as an RPW dataset. An asterisk (*) indicates that public availability on Figshare is reported in the original study [40].

**Table 9:** Principal benchmarking and reproducibility challenges in RPW detection research.

Challenge	Manifestation in the Reviewed Literature	Consequence	Recommended Remedy
Dataset-provenance and species mismatch	Cross-species corpora (e.g., TreeVibes) reused without clear insect and host provenance	Results may be misinterpreted as RPW-specific validation	Report insect and host species, site, and biological verification; label proxy data explicitly
Heterogeneous data partitions	Train–validation–test ratios and cross-validation schemes vary across studies	Results are not directly comparable	Use fixed public training, validation, and untouched test partitions
Data-leakage risk	Sample-level splits of recordings, signal segments, frames, or image crops may place data from the same palm, session, or site in multiple partitions	Tree- or session-specific patterns may inflate performance	Use palm-, session-, farm-, or site-level grouped splitting; report the grouping unit
Inconsistent metric reporting	Accuracy emphasised; recall, specificity, PR-AUC, false-negative and false-alarm rates reported inconsistently	Class imbalance and missed infestations may be concealed	Report precision, recall, F1, specificity, ROC-AUC, PR-AUC, false-negative and false-alarm rates with confidence intervals
Class-prevalence effects	Class distributions differ while accuracy and PR-AUC are compared without reporting prevalence	Differences may reflect class composition rather than classifier quality	Report class counts and prevalence; interpret ROC-AUC and PR-AUC jointly [63,64]
Private dataset and code dominance	Raw data, annotations, scripts, weights, and configurations frequently unavailable	Independent replication prevented	Deposit data, code, weights, splits, and configurations in persistent FAIR repositories where ethically and legally permissible [65]
Incomplete environmental metadata	Temperature, humidity, wind, acquisition time, season, and sensor position inconsistently documented	Environmental degradation and domain shift cannot be modelled	Define mandatory environmental and sensor-acquisition metadata fields
Limited infestation-stage annotation	Binary healthy/infested labels predominate without validated severity or developmental-stage labels	Early-stage sensitivity and intervention timing cannot be evaluated	Develop expert-validated early-, intermediate-, and advanced-stage annotation protocols
Absence of synchronised multimodal data	No identified benchmark within the reviewed corpus combines time-aligned acoustic, thermal, RGB, and environmental measurements from the same palms	Multimodal fusion cannot be evaluated fairly against unimodal baselines	Acquire co-registered, timestamped multimodal observations with shared palm identifiers
Geographic and biological concentration	Data originate from a limited range of countries, climates, cultivars, and RPW populations	Models may fail under new conditions	Require multi-site, multi-season, multi-cultivar, and cross-country external validation
No shared evaluation framework	No common baseline suite, evaluation toolkit, or leaderboard exists	Dataset novelty confounded with algorithmic improvement	Establish a shared, versioned benchmark with public baselines, hidden test data, and a transparent leaderboard
Pseudo-replication risk	Large numbers of windows/crops extracted from few independent palms or sessions	Inflated sample size and over-optimistic performance estimates	Report number of independent palms, sites, and sessions alongside segment/image counts

**Table 10:** Cross-technology comparison of operational characteristics and reported evidence across RPW detection paradigms.

Approach	Primary Operational Target	Earliest Demonstrated Evidence	Reported Outcome or Evidence Basis	Relative Cost	Scalability	Automation	Principal Limitation	Representative Sources
Visual inspection	Identification of symptomatic palms	Externally visible symptoms after infestation is established	Routine operational surveillance; no transferable accuracy estimate	Low	Low	None	Poor sensitivity to concealed and early internal infestation	[1,22]
Pheromone trapping	Adult population and area-level surveillance	Adult activity only	Trap counts indicate adult activity but cannot identify an individual infested palm	Low	High	Partial	Cannot detect eggs, larvae, or pupae within a specific palm	[8,24]
Canine olfactory detection	Targeted screening for RPW-associated odour	Concealed adults or larvae in experimentally baited palms	Approximately 78% true-positive indications and 12.6% false-positive indications were reported in experimentally baited Canary Island date palms; diagnostic performance under naturally infested field conditions remains unestablished.	High	Low	None	Artificial-baiting evidence, handler dependence, fatigue, training cost, variable performance, and uncertain sensitivity under natural early infestation	[25,26]
Contact acoustic sensing	Tree-level detection of larval feeding and movement	Active larval infestation; weakest during the earliest phase	Stage-dependent sensitivity (e.g., averaged ~80%, peaked ~95%, fell to ~33% in early infestation in one study)	Medium	Medium	Partial	Sensitive to larval activity, background noise, sensor coupling, palm age, and infestation stage	[4,29]
Networked IoT vibroacoustics	Continuous remote monitoring of internal borer activity	Active internal borer vibration	High reported accuracy in subsequent TreeVibes-based studies, but the benchmark is multi-species and not RPW-only	Medium	High	High	Host- and species-domain mismatch; limited generalisation to RPW-only deployment	[34,37,54]
Thermal imaging and thermal AI	Detection of thermal anomalies associated with tissue damage	Established infestation or severe physiological effects	Single-site study reported 98.5% accuracy on 6000 thermal images; no external validation	Medium	Medium	High	Thermal contrast varies with solar radiation, temperature, humidity, acquisition time, and tissue depth	[42]
UAV RGB–multispectral–thermal sensing	Orchard-scale identification of anomalous palms	Severe infestation before visible canopy symptoms	Severe-stage thermal and spectral separation demonstrated; no validated classifier performance	High	High	High	Early and moderate stages not demonstrated; requires repeated flights, registration, and strong biological ground truth	[12]
Fibre-optic DAS	Distributed monitoring of larval vibration	Approximately 12-day-old larvae in controlled experiments	Approximately 97% signal classification in limited experiments; outdoor performance sensitive to wind	High	High	High	Infrastructure cost, installation complexity, wind sensitivity, and absence of multi-farm validation	[10,16,32]
Microwave sensing	Detection of dielectric or scattering differences in palm tissue	Analytical or phantom-based dielectric contrast; no biologically verified infestation stage	Analytical or simulation-based evidence only; no intact-palm orchard validation	High	Medium	High	Simplified models, uncertain dielectric properties, clutter, penetration–resolution trade-off	[14,15]
Multimodal AI fusion	Integrated palm-level classification using complementary modalities	Potentially multi-stage	High study-specific results, but no synchronised multimodal, multi-site benchmark	High	Medium	High	No synchronised benchmark, limited ablation analysis, and no multi-site integrated validation	[56,59,61,62]

Note. Reported outcomes are reproduced as presented in the source studies and are not directly comparable across technologies because the underlying tasks, sampling units, datasets, infestation stages, and validation designs differ. Relative cost, scalability, and automation are review-assigned comparative judgments rather than formal economic or deployment assessments. Operational maturity and technology readiness are assessed separately in Table 11.

**Table 11:** Review-assigned indicative operational maturity and technology readiness for RPW detection and surveillance approaches.

Approach	Indicative Maturity/TRL	Interpretive Criterion	Evidence Mapping	Confidence	Required Milestone
Visual inspection	Operationally established	Established surveillance practice in the intended operational environment	Routine national and regional surveillance use	High	Digitise records; standardise symptoms and inspection; integrate sensor screening
Pheromone trapping	Operationally established	Established population-monitoring practice in the intended operational environment	Widely used for adult-population monitoring	High	Automate traps; optimise placement; integrate risk maps
Canine olfactory detection	TRL 4–5	Component validated in laboratory or limited relevant environments	Feasibility demonstrated in experimentally baited palms; diagnostic performance under naturally infested field conditions remains unconfirmed [25,26]	Moderate–low	Blinded multi-site, multi-handler trials with standard diagnostic metrics
Contact acoustic sensing	TRL 5–6	Component validated and prototype partly demonstrated in a relevant environment	Stage-dependent acoustic detection demonstrated in naturally infested young palms and commercial orchard environments; automated detection was feasible under field noise, but standardised multi-site operational validation remains limited [4,29,33]	Moderate	Standardise sensors and protocols; validate across commercial plantations
Networked IoT vibroacoustics—RPW application	TRL 4–5	Technology component validated in a relevant borer-monitoring environment	Remote acquisition and automated borer classification shown, mainly outside RPW orchards [30,34,54]	Moderate–low	Multi-farm RPW-only validation across cultivars, seasons, and environments
Thermal imaging and thermal AI	TRL 5	Technology component and prototype validated under relevant field-derived conditions	Thermal sensing has been evaluated in commercial and field environments, while thermal-AI and edge implementations have been demonstrated using field-derived imagery; integrated multi-site operational prototype validation remains lacking [5,39,40,42].	Moderate	Multi-site, multi-season testing with confirmed infestation stages and independent farms
UAV RGB–multispectral–thermal sensing	TRL 5	Technology validated in a relevant experimental field environment	Two-year UAV monitoring of experimentally infested date palms demonstrated thermal and spectral separation under field conditions, including differences before visible canopy symptoms; validation remained single-site and proof-of-concept [12]	Moderate	Validate commercial-orchard classifiers by confirmed infestation stage
Fibre-optic DAS	TRL 5	Technology component validated in controlled and limited relevant environments	Initial laboratory feasibility was demonstrated using playback of recorded sounds from young RPW larvae within a reconstructed farm environment that included simulated wind and bird noise [10]. Subsequent studies evaluated fibre-optic DAS using live RPW larvae under controlled conditions [32] and in a limited outdoor experiment involving two artificially infested palms [16]. Multi-farm validation was not reported.	Moderate	Multi-tree, multi-farm trials; quantify cost and wind robustness
Microwave sensing	TRL 3–4	Technology concept formulated and analytical proof-of-concept demonstrated	Evidence spans theoretical modelling and laboratory validation using cut palm trunks with RPW pupal phantoms; validation on live, naturally infested palms or in orchards has not been demonstrated [14,15]	Low	Validate calibrated microwave hardware on live, naturally infested palms; quantify penetration depth, localisation accuracy, SNR, and biological ground truth under orchard conditions.
Multimodal AI fusion	TRL 4–5	Multimodal components validated on experimental or field-derived data, with limited field demonstration.	Evidence spans computational fusion and limited field-based acoustic–visual workflows; acoustic–microwave fusion used non-simultaneous field acoustic and laboratory microwave data. No synchronised, independently validated multi-site multimodal RPW system has yet been demonstrated [56,59,60,61,62]	Moderate–low	Validate synchronised, co-registered multimodal sensing on the same palms across independent orchards, with matched unimodal baselines and modality-ablation analysis.

Note. Operational-maturity descriptors are used for established surveillance practices such as visual inspection and pheromone trapping. Numeric TRL values are reserved for engineered RPW-specific sensing and AI systems and refer to the maturity of the integrated approach rather than individual sensors, algorithms, or commercial components. All values are review-assigned indicative judgments and should not be interpreted as independently validated or formally certified readiness levels.

**Table 12:** Research gap summary—evidence base, operational impact, and proposed research directions.

Research Gap	Evidence from the Reviewed Literature	Operational Implication	Priority Research Direction
RG-1: Reliable early-stage detection	Current systems generally require measurable larval activity, tissue damage, or physiological stress	Treatable infestations may remain undetected until damage becomes established	Evaluate high-sensitivity vibroacoustics, VOC sensing, and experimentally validated microwave or dielectric methods on palms with biologically verified infestation stage and independent ground truth
RG-2: RPW-native benchmark infrastructure	Available datasets are fragmented, geographically restricted, and sometimes contain cross-species borer recordings; synchronised multimodal data remain unavailable	Results cannot be reproduced or compared fairly across studies	Establish a FAIR, multi-site, multi-season RPW benchmark with persistent palm identifiers, synchronised modalities, versioned public development partitions, reproducible baselines, and, where feasible, independently administered hidden testing
RG-3: Prospective multi-site validation	Much of the evidence is based on controlled experiments, artificial infestation, single sites, or retrospective datasets	Reported performance may not generalise to commercial plantations	Conduct blinded multi-site validation across farms, cultivars, climates, seasons, and sensor installations using independent external test sets
RG-4: Explainability and accountability	Recognised XAI methods were rarely applied, and quantitative evaluation of explanation faithfulness, stability, or decision utility was not identified	False alarms and missed infestations may be difficult to justify in phytosanitary decisions	Integrate modality-specific explanations and evaluate their faithfulness, stability, and agronomic interpretability
RG-5: Validated multimodal fusion	Existing fusion studies frequently use independently acquired modality datasets, loosely aligned observations, or decision-level combination	The true contribution of modality complementarity remains uncertain	Collect synchronised acoustic, thermal, visual, and environmental data from the same palms and compare fusion architectures against matched unimodal baselines
RG-6: Weather-aware and domain-adaptive inference	Environmental variables are usually treated as post hoc limitations rather than operational inputs	Wind, temperature, illumination, and seasonal variation may cause domain shift and unstable predictions	Develop environmentally conditioned thresholds, quality-control gates, adaptive scheduling, uncertainty-aware rejection, and domain-adaptation methods; monitor calibration drift across sensors and sites
RG-7: Edge-AI and resource-efficiency benchmarking	Edge suitability is often claimed without systematic embedded-hardware evaluation	Practical deployment remains uncertain because latency, energy, memory, and communication requirements are insufficiently quantified	Benchmark complete sensing pipelines on representative edge platforms using latency, energy, memory, throughput, communication load, and post-quantisation accuracy
RG-8: Stage- and severity-aware diagnosis	Binary healthy–infested labels dominate, while validated progression and severity labels remain uncommon	Detection outputs provide limited guidance for treatment selection or palm removal	Develop entomologically and agronomically validated ordinal severity labels and longitudinal datasets linking infestation progression with sensor responses

## Data Availability

The methodological appraisal and search documentation are provided in Supplementary Tables S1 and S2, respectively. The retrospective review protocol, a machine-readable title/abstract screening log derived from the Rayyan (https://www.rayyan.ai/, accessed on 30 September 2026) screening export, and the supplementary workbook containing Tables S1 and S2 are publicly available on the Open Science Framework at https://osf.io/c2kv5/ (accessed on 20 September 2026). The completed PRISMA 2020 checklist is provided as Supplementary File S1.

## Supplementary Materials

The following supporting information can be downloaded at https://www.mdpi.com/article/10.3390/insects17101081/s1, Table S1. Methodological reporting and validation appraisal of the 56 included studies: References [4,5,8,10,11,12,13,14,15,16,23,25,26,27,28,29,30,31,32,33,34,35,36,37,38,39,40,42,43,44,45,46,47,48,49,50,51,52,53,54,55,56,57,58,59,60,61,62,73,74,75,76,77,78,79,80]; Table S2. Database-specific search strategies and supplementary search documentation; Supplementary File S1. PRISMA 2020 Checklist; Supplementary File S2: RPW_Rayyan_Screening_Log_123_Records.

## Abbreviations

The following abbreviations are used in this manuscript:

AI	Artificial intelligence
ANN	Artificial neural network
BFCC	Bark-frequency cepstral coefficients
BSA	Bird swarm algorithm
CNN	Convolutional neural network
CQCC	Constant-Q cepstral coefficients
DAS	Distributed acoustic sensing
DL	Deep learning
FAIR	Findable, Accessible, Interoperable, and Reusable
FDTD	Finite-difference time-domain
GIS	Geographic information system
GLCM	Grey-level co-occurrence matrix
Grad-CAM	Gradient-weighted class activation mapping
GSM	Global System for Mobile Communications
GTO	Gorilla Troops Optimizer
IoT	Internet of Things
LBP	Local binary pattern
LFCC	Linear-frequency cepstral coefficients
LoRa	Long Range
LPC	Linear predictive coding
LSTM	Long short-term memory
LWIR	Long-wave infrared
MAML	Model-agnostic meta-learning
MEMS	Microelectromechanical systems
MFCC	Mel-frequency cepstral coefficients
ML	Machine learning
MLP	Multilayer perceptron
NR	Not reported
ORB	Oriented FAST and rotated BRIEF
PR-AUC	Area under the precision–recall curve
PRISMA	Preferred Reporting Items for Systematic Reviews and Meta-Analyses
R-CNN	Region-based convolutional neural network
RF	Random forest
RGB	Red–green–blue
RNN	Recurrent neural network
ROC-AUC	Area under the receiver operating characteristic curve
RPW	Red Palm Weevil
SHAP	SHapley Additive exPlanations
SIFT	Scale-invariant feature transform
SNN	Spiking neural network
SNR	Signal-to-noise ratio
SVM	Support vector machine
TIR	Thermal infrared
TRL	Technology readiness level
UAV	Unmanned aerial vehicle
ViT	Vision Transformer
VOC	Volatile organic compound
XAI	Explainable artificial intelligence
XGBoost	Extreme Gradient Boosting
YOLO	You Only Look Once
Φ-OTDR	Phase-sensitive optical time-domain reflectometry
