# Supplementary material for: Early Detection of Red Palm Weevil Infestation: A Systematic Review of Sensing Technologies, Artificial Intelligence, and Field Readiness
Source: Insects. 2026 Oct 19;17(10):1081. doi: 10.3390/insects17101081 (PMC13705317; doi:10.3390/insects17101081)
Supplement: Supplementary file 1 [file insects-17-01081-s001.zip › Supplementary_File_S1_PRISMA_2020_Checklist.pdf]

Manuscript: Early Detection of Red Palm Weevil Infestation: A Systematic Review of Sensing Technologies, Artificial Intelligence, and Field Readiness

Supplementary File S1. PRISMA 2020 Checklist

Section and Topic	Item #	Checklist item	Location where item is reported
**TITLE**			
Title	1	Identify the report as a systematic review.	Title page: the manuscript title identifies the report as a systematic review.
**ABSTRACT**			
Abstract	2	See the PRISMA 2020 for Abstracts checklist.	Abstract: review objective, evidence base, principal findings and limitations. Funding and retrospective protocol details appear under Funding and Section 2.1, respectively; they are not included in the abstract.
**INTRODUCTION**			
Rationale	3	Describe the rationale for the review in the context of existing knowledge.	Sections 1.1–1.3: importance of the pest, early-detection problem, biological basis, and the development of detection technologies.
Objectives	4	Provide an explicit statement of the objective(s) or question(s) the review addresses.	Section 1.4, Objectives and Contributions of This Review; Section 2.1, research questions.
**METHODS**			
Eligibility criteria	5	Specify the inclusion and exclusion criteria for the review and how studies were grouped for the syntheses.	Sections 2.1 and 2.3; Table 1 (IC1–IC6 and EC1–EC6). Sections 2.4–2.5 and the modality-based organisation of Sections 3–9 describe grouping for synthesis.
Information sources	6	Specify all databases, registers, websites, organisations, reference lists and other sources searched or consulted to identify studies. Specify the date when each source was last searched or consulted.	Section 2.2; Supplementary Table S2, Sections A–D. Initial searches are dated May 2026; the supplementary update, source-verification documentation and targeted canine check are distinguished.
Search strategy	7	Present the full search strategies for all databases, registers and websites, including any filters and limits used.	Section 2.2; Supplementary Table S2, Sections A–D, including source-specific queries, filters and documentation limitations. Queries not retained for some source-verification exports are explicitly identified in S2.
Selection process	8	Specify the methods used to decide whether a study met the inclusion criteria of the review, including how many reviewers screened each record and each report retrieved, whether they worked independently, and if applicable, details of automation tools used in the process.	Section 2.3: two reviewers independently screened titles/abstracts and full texts; disagreements were resolved by consensus. Section 2.5 describes the limits of AI assistance.

PRISMA

PRISMA 2020 Checklist

Section and Topic	Item #	Checklist item	Location where item is reported
Data collection process	9	Specify the methods used to collect data from reports, including how many reviewers collected data from each report, whether they worked independently, any processes for obtaining or confirming data from study investigators, and if applicable, details of automation tools used in the process.	Section 2.4: structured extraction, Elicit-assisted preliminary extraction and manual verification against the original publications. Independence of study-data extraction and investigator-contact procedures are not explicitly reported; reviewer checking is specified for review-level coding.
Data items	10a	List and define all outcomes for which data were sought. Specify whether all results that were compatible with each outcome domain in each study were sought (e.g. for all measures, time points, analyses), and if not, the methods used to decide which results to collect.	Section 2.4 lists performance metrics, targeted infestation stage and validation environment. Rules for selecting among multiple compatible results within each outcome domain are not explicitly reported.
	10b	List and define all other variables for which data were sought (e.g. participant and intervention characteristics, funding sources). Describe any assumptions made about any missing or unclear information.	Section 2.4 lists bibliographic, method, model, sensor, dataset and explainability variables, together with review-assigned classifications. Sections 2.4–2.5 describe NR for missing or insufficient evidence.
Study risk of bias assessment	11	Specify the methods used to assess risk of bias in the included studies, including details of the tool(s) used, how many reviewers assessed each study and whether they worked independently, and if applicable, details of automation tools used in the process.	Section 2.4; Supplementary Table S1. The five-item appraisal assesses reporting completeness and selected validation features; it is explicitly not a formal risk-of-bias assessment.
Effect measures	12	Specify for each outcome the effect measure(s) (e.g. risk ratio, mean difference) used in the synthesis or presentation of results.	Sections 2.4–2.5: study-reported performance measures are extracted; no common effect measure is available for pooling. Study-specific measures appear in Tables 2–8.
Synthesis methods	13a	Describe the processes used to decide which studies were eligible for each synthesis (e.g. tabulating the study intervention characteristics and comparing against the planned groups for each synthesis (item #5)).	Sections 2.1 and 2.3–2.5; Table 1. Sections 3–9 organise eligible evidence by detection modality, biological task, validation context and comparative question.
	13b	Describe any methods required to prepare the data for presentation or synthesis, such as handling of missing summary statistics, or data conversions.	Sections 2.4–2.5: missing or unverifiable information is recorded as NR rather than inferred. Review-assigned classifications are distinguished from study-reported outcomes.
	13c	Describe any methods used to tabulate or visually display results of individual studies and syntheses.	Section 2.5 describes narrative analysis and comparative tables. Tables 2–12 present study-level and cross-technology comparisons; Figures 4–5 present the conceptual framework and stage-oriented evidence synthesis.
	13d	Describe any methods used to synthesize results and provide a rationale for the choice(s). If meta-analysis was performed, describe the model(s), method(s) to identify the presence and extent of statistical heterogeneity, and software package(s) used.	Section 2.5: narrative synthesis and comparative tables. Differences in methods, biological targets, validation and outcomes preclude meta-analysis; no common effect measure is available.

PRISMA 2020 Checklist

Section and Topic	Item #	Checklist item	Location where item is reported
	13e	Describe any methods used to explore possible causes of heterogeneity among study results (e.g. subgroup analysis, meta-regression).	Sections 2.5, 8 and 9 discuss heterogeneity descriptively. Formal subgroup analysis or meta-regression is not reported.
	13f	Describe any sensitivity analyses conducted to assess robustness of the synthesized results.	No sensitivity-analysis method is reported.
Reporting bias assessment	14	Describe any methods used to assess risk of bias due to missing results in a synthesis (arising from reporting biases).	A formal assessment of bias due to missing results is not reported.
Certainty assessment	15	Describe any methods used to assess certainty (or confidence) in the body of evidence for an outcome.	A formal certainty-of-evidence framework is not reported. Section 2.4 describes review-level evidence-strength and maturity coding.
**RESULTS**			
Study selection	16a	Describe the results of the search and selection process, from the number of records identified in the search to the number of studies included in the review, ideally using a flow diagram.	Section 2.3; Figure 3; Supplementary Table S2, Section E. The final corpus comprises 51 studies from the initial search, four supplementary additions and one targeted canine-detection addition (56 studies).
	16b	Cite studies that might appear to meet the inclusion criteria, but which were excluded, and explain why they were excluded.	Figure 3 and Supplementary Table S2, Section F report aggregate reasons for 44 full-text exclusions. Individual citations and exclusion reasons for all apparently eligible excluded reports are not presented.
Study characteristics	17	Cite each included study and present its characteristics.	Supplementary Table S1 identifies the 56 included studies. Sections 3–8 and Tables 2–8 present study characteristics by modality and biological task.
Risk of bias in studies	18	Present assessments of risk of bias for each included study.	Section 2.4 and Supplementary Table S1 present study-level reporting/validation appraisal results (40 high, 15 moderate and one low score). These scores are not formal risk-of-bias ratings.
Results of individual studies	19	For all outcomes, present, for each study: (a) summary statistics for each group (where appropriate) and (b) an effect estimate and its precision (e.g. confidence/credible interval), ideally using structured tables or plots.	Sections 3–8 and Tables 2–8 report study-specific performance and validation information. Precision estimates are not consistently presented.
Results of syntheses	20a	For each synthesis, briefly summarise the characteristics and risk of bias among contributing studies.	Sections 3–9 summarise study characteristics, reported outcomes and limitations. Section 2.5 explains how appraisal findings inform interpretation; Supplementary Table S1 contains individual appraisal results. No formal risk-of-bias synthesis is reported.

PRISMA 2020 Checklist

Section and Topic	Item #	Checklist item	Location where item is reported
	20b	Present results of all statistical syntheses conducted. If meta-analysis was done, present for each the summary estimate and its precision (e.g. confidence/credible interval) and measures of statistical heterogeneity. If comparing groups, describe the direction of the effect.	No pooled statistical synthesis or meta-analysis was performed (Section 2.5). Results are synthesised narratively and in comparative tables.
	20c	Present results of all investigations of possible causes of heterogeneity among study results.	Sections 8–9 discuss differences in datasets, tasks, validation and environmental conditions narratively. Formal statistical investigations of heterogeneity are not reported.
	20d	Present results of all sensitivity analyses conducted to assess the robustness of the synthesized results.	No sensitivity-analysis results are reported.
Reporting biases	21	Present assessments of risk of bias due to missing results (arising from reporting biases) for each synthesis assessed.	Formal reporting-bias assessment results are not reported.
Certainty of evidence	22	Present assessments of certainty (or confidence) in the body of evidence for each outcome assessed.	Formal certainty-of-evidence assessment results are not reported.
**DISCUSSION**			
Discussion	23a	Provide a general interpretation of the results in the context of other evidence.	Section 9, Comparative Synthesis, particularly Section 9.4; Section 11, Conclusion.
	23b	Discuss any limitations of the evidence included in the review.	Sections 8–9, particularly Section 9.4; Tables 9–11: dataset limitations, heterogeneous outcomes and validation, environmental robustness, and evidence boundaries.
	23c	Discuss any limitations of the review processes used.	Section 9.4: language and full-text eligibility restrictions, retrospective protocol recording, search reproducibility, incomplete rerunning of original sources, and the limits of the review-specific appraisal.
	23d	Discuss implications of the results for practice, policy, and future research.	Section 10 and Table 12 set out research priorities and operational implications; Section 11 presents conclusions and recommendations.
**OTHER INFORMATION**			
Registration and protocol	24a	Provide registration information for the review, including register name and registration number, or state that the review was not registered.	Section 2.1: the review was not prospectively registered. A retrospective, time-stamped OSF protocol record was deposited on 5 August 2026 (reference [20]).
	24b	Indicate where the review protocol can be accessed, or state that a protocol was not prepared.	Section 2.1 and reference [20]: https://doi.org/10.17605/osf.io/fds69. The retrospective protocol is also available through the OSF project identified in the Data Availability Statement: https://osf.io/c2kv5/.

PRISMA 2020 Checklist

Section and Topic	Item #	Checklist item	Location where item is reported
	24c	Describe and explain any amendments to information provided at registration or in the protocol.	Sections 2.2–2.3 and Supplementary Table S2, Section D describe the targeted canine-detection addition during revision. A formal amendment log against the retrospective protocol is not presented.
Support	25	Describe sources of financial or non-financial support for the review, and the role of the funders or sponsors in the review.	Funding statement: Princess Nourah bint Abdulrahman University, Supporting Publication in Review Article Program, grant RAP-2026-03. The funder's role is not explicitly stated.
Competing interests	26	Declare any competing interests of review authors.	Conflicts of Interest statement: the authors declare no conflict of interest.
Availability of data, code and other materials	27	Report which of the following are publicly available and where they can be found: template data collection forms; data extracted from included studies; data used for all analyses; analytic code; any other materials used in the review.	Data Availability Statement; Supplementary Materials. S1: methodological appraisal; S2: search documentation; S3: the completed PRISMA 2020 checklist. The retrospective protocol, screening log and S1/S2 workbook are available at https://osf.io/c2kv5/.

From: Page MJ, McKenzie JE, Bossuyt PM, Boutron I, Hoffmann TC, Mulrow CD, et al. The PRISMA 2020 statement: an updated guideline for reporting systematic reviews. BMJ 2021;372:n71. doi: 10.1136/bmj.n71.
This work is licensed under CC BY 4.0. To view a copy of this license, visit https://creativecommons.org/licenses/by/4.0/
